# Disentangling single-cell omics representation with a power spectral density-based feature extraction

**DOI:** 10.1093/nar/gkac436

**Published:** 2022-05-25

**Authors:** Seid Miad Zandavi, Forrest C Koch, Abhishek Vijayan, Fabio Zanini, Fatima Valdes Mora, David Gallego Ortega, Fatemeh Vafaee

**Affiliations:** School of Biotechnology and Biomolecular Sciences, University of New South Wales (UNSW Sydney), Australia; Programs in Metabolism and Medical & Population Genetics, Broad Institute, Cambridge, MA, USA; Division of Genetics and Genomics, Boston Children's Hospital, Boston, MA, USA; Department of Pediatrics, Harvard Medical School, Boston, MA, USA; School of Biotechnology and Biomolecular Sciences, University of New South Wales (UNSW Sydney), Australia; School of Biotechnology and Biomolecular Sciences, University of New South Wales (UNSW Sydney), Australia; Prince of Wales Clinical School, UNSW Sydney, Australia; Cellular Genomics Future Institute, UNSW Sydney, Australia; Children's Cancer Institute, Lowy Cancer Research Centre, UNSW Sydney, Australia; School of Women's and Children's Health, Faculty of Medicine, UNSW, Sydney, Australia; School of Biomedical Engineering, University of Technology Sydney (UTS), Australia; School of Biotechnology and Biomolecular Sciences, University of New South Wales (UNSW Sydney), Australia; Cellular Genomics Future Institute, UNSW Sydney, Australia; UNSW Data Science Hub (uDASH), UNSW Sydney, Australia

## Abstract

Emerging single-cell technologies provide high-resolution measurements of distinct cellular modalities opening new avenues for generating detailed cellular atlases of many and diverse tissues. The high dimensionality, sparsity, and inaccuracy of single cell sequencing measurements, however, can obscure discriminatory information, mask cellular subtype variations and complicate downstream analyses which can limit our understanding of cell function and tissue heterogeneity. Here, we present a novel pre-processing method (scPSD) inspired by *power spectral density* analysis that enhances the accuracy for cell subtype separation from large-scale single-cell omics data. We comprehensively benchmarked our method on a wide range of single-cell RNA-sequencing datasets and showed that scPSD pre-processing, while being fast and scalable, significantly reduces data complexity, enhances cell-type separation, and enables rare cell identification. Additionally, we applied scPSD to transcriptomics and chromatin accessibility cell atlases and demonstrated its capacity to discriminate over 100 cell types across the whole organism and across different modalities of single-cell omics data.

## INTRODUCTION

Continuous innovations in single-cell technologies allow the interrogation of a growing number of molecular modalities such as DNA, chromatin, mRNA and protein, at high-resolution and across thousands of cells from complex biological systems. Increased throughput of new single-cell technologies has posed unique analytical challenges demanding for scalable computational methods that can analyze diverse high-dimensional omics data highly accurately and fast (1). Single-cell sequencing data also suffer from the ‘curse of missingness’ due to, for instance, dropout events in scRNA-sequencing (2) or the low copy number in DNA leading to an inherent per-cell sparsity in scATAC-sequencing data (3). High-dimensionality and sparsity, combined with various systematic biases in single-cell sequencing experiments (4), obscure important information in data which hinders precise distinctions among cell states and masks shared biological signals among different cell subtypes. Extracting discriminatory information is therefore essential for the success and accuracy of downstream analyses and is particularly relevant for the application of machine learning methods to diverse problems from cell-type classification to trajectory inference or multimodal data integration (5).

Feature extraction seeks an optimal transformation of the input data into a latent feature vector with the primary goal of extracting important information from input data, controlling for confounding effects, adjusting over-dispersion, and removing redundancy to enhance the separation of distinct cellular phenotypes (6). Dimensionality reduction (DR) techniques such as PCA (principal component analysis) (7), t-SNE (t-distributed stochastic neighbor embedding) (8), and UMAP (Uniform Manifold Approximation and Projection) (9) are frequently employed to transform high-dimensional data into a low-dimensional space, which is particularly useful to visually inspect the distribution of input data. Further feature extraction methods were specifically developed for scRNA-sequencing data – e.g. ZIFA (zero inflated factor analysis) (10), ZinbWave (zero-inflated negative binomial model) (11), and scVI (single-cell variational inference) (12) – or to a much lesser extent, for other single-cell modalities—e.g. SCALE (single-cell ATAC-seq analysis via latent feature extraction) (13). DR methods have varied performance in separating biological clusters as per our recent comprehensive benchmarking (14) and often perform poorly in facilitating the detection of rare cell populations (15). Furthermore, the capacity of different DR methods in extracting features from other single-cell omics, beyond scRNA-sequencing data, is undetermined and yet to be assessed systematically.

**Figure 1.**
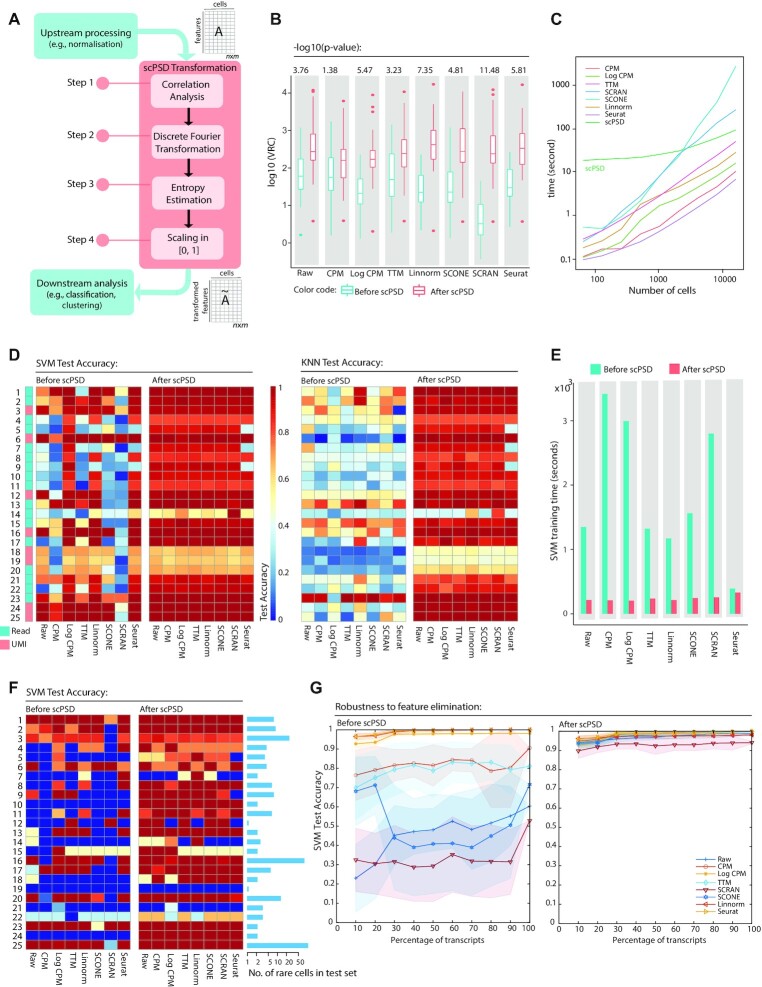
Overview of scPSD and performance evaluation on scRNA-seq datasets. (**A**) the scPSD transformation framework comprising four consecutive steps of feature extraction and standardization. scPSD can fit into a single-cell sequencing analysis pipeline after the upstream processing (or directly on raw data) to enhance downstream analyses. (**B**) box plots comparing VRC (variance ratio criterion) as a measure of how well-formed distinct cell-types are before/after scPSD transformation of normalized and raw counts across 25 curated scRNA-seq datasets (numbered according to Supplementary Table S1); *P*-values of t-tests comparing log_10_-transformed VRC measures before and after scPSD are reported on top of each pair of boxplots. SS (silhouette score) and mFDR (multi-class Fisher's discriminant ratio) as other measures of cluster separation and dataset complexity are reported in Supplementary Figure S2. (**C**) computational runtime of scPSD and normalization methods as scales with increasing number of cells. Methods were applied to datasets of varying size obtained by random subsampling of the 10X Genomics E18 mouse dataset, and timings are averaged over 16 applications. (**D**) heatmaps representing accuracy of cell-type prediction—for each of 25 scRNA-seq datasets—on 20% randomly held out data (test set) after training SVM (support vector machine) and KNN (k-nearest neighbor) models on remaining 80% of data (training set), before and after scPSD transformation. (**E**) SVM training time in second before and after scPSD transformation demonstrating significant reduction in convergence time after transformation. (**F**) heatmaps representing SVM test accuracy identifying rare cell-type identification—defined as the smallest cell-type population constituting <1% to 14% of captured cells across 25 scRNA-seq datasets—before and after scPSD transformation. (**G**) SVM test accuracy upon increasing feature coverage using ‘deng reads’ dataset (#13 in Supplementary Table S1). The procedure includes random accumulation of genes (in 10% brackets), reporting SVM test accuracy (on 20% holdout cells) before and after scPSD transformation, and repeating the procedure 100 times to account for random feature selection. The average trends were reported with shades representing ± standard deviation across 100 repeats.

Here, we present an innovative unified strategy for single-cell omics data transformation (scPSD) that is inspired by *power spectral density* (PSD) analysis (16) to intensify discriminatory information from single-cell genomic features. PSD is a statistical signal processing technique to describe the distribution of power over frequency and to show the strength of the energy as a function of frequency (16). One purpose of estimating spectral density is to detect any patterns or periodicities in a signal by observing peaks at the frequencies corresponding to these patterns. Here, a vector of genomic features (e.g. expressions of transcripts, open chromatin regions, or cell-surface proteins in a single cell) has been realized as a ‘signal’ representing a cellular state. The scPSD feature transformation performs four consecutive steps on ‘single-cell genomic signals’ (Figure 1A):

(i) Estimating pairwise correlations of genomic features across cells followed by within-cell correlation mapping. (ii) Feature extraction by discrete Fourier transformation (DFT), a mathematical approach widely used to reveal hidden patterns and periodicities across a finite data sequence upon transformation into the frequency domain. As reviewed elsewhere (6), DFT has been used in a variety of bioinformatics applications for the analysis of repetitive elements in DNA sequences and protein structures, among others. We implemented the fast Fourier transform (FFT), a highly efficient procedure for computing the DFT of a data sequence (17). (iii) Entropy estimation to improve the extraction of important information from Fourier transformed data. We employed Shannon's entropy (18) which describes the uncertainty in discrete random variables representing the information content of a probabilistic event. Entropy-based methods have been frequently used for feature extraction and analysis of biological sequences as reviewed previously (19,20). (iv) Scaling transformed values between zero and one.

The scPSD transformation can fit into any single-cell computational pipeline complementing the upstream pre-processing (e.g. normalization) to improve data quality, and streamlining downstream computations (Figure 1A) as demonstrated by extensive analyses presented in this study.

## MATERIALS AND METHODS

### Overview of scPSD

A signal can be considered as a series of measurements that conveys information about the behavior of a system. Inspired by the idea that a cell is a biological system whose behavior can be realized by a collective quantification of pools of molecules (i.e. omics), we considered an ‘omics signal’ of length $n$, denoted $a\ = ( {{a_1},{a_2}, \ldots ,{a_n}} )\ \in {\mathbb{R}^n}$, as a series of molecular measurements (ordered in any random arrangement).

Any signal can be decomposed into a number of discrete frequencies according to Fourier analysis. The statistical average of the signal in terms of its frequency content is called its spectrum which often contains essential information about the nature of the signal and behavior of the system. The power spectral density (PSD), or simply power spectrum, describes the distribution of *energy* into frequency components composing a signal where energy is defined as the area under the squared magnitude of the considered signal (16). Power spectral density, therefore, indicates energetic frequencies to extract patterns and periodicities of signal. The power spectral density can be found as the Fourier transform of the autocorrelation function (21). Autocorrelation is the correlation of a signal with a delayed copy of itself as a function of delay and depends on the ordering of datapoints in a series. However, molecular measurements (omics) are often arbitrarily ordered (e.g. genes in transcriptomic profiles). We, therefore, relaxed the ordering dependency by estimating pairwise correlations among all features (e.g. genes) across samples. The ‘omics signal’ was linearly transformed by the correlation matrix to reflect cross-sample dependencies and undergone Fourier transformation whose magnitude represents the amount of ‘power’ per unit of the signal. The resultant ‘power spectrum’ were then used to estimate the ‘spectral entropy’ (22) describing the irregularity of the power distribution as an indication of the complexity of a *system* (i.e. a *cell* in this context). Finally, the entropy-based transformed measures were normalised between zero and one resulting final latent features for downstream analyses. Accordingly, scPSD implements the following four consecutive steps (after filtering genes with zero expression across all cells):

#### Step 1. Correlation estimation

Let's denote a single cell omics dataset as a matrix $A\ = ( {{a_{kj}}} )\ \in {\mathbb{R}^{n \times m}}$ of $n$ measurements (e.g. genes) and $m$ samples (i.e. cells). Across-sample correlation is obtained by computing pairwise linear correlation coefficient between each pair of genes: $\rho \ = {\rm{corr}}( {{A^T}} )\ = ( {{\rho _{kj}}} )\ \in {\mathbb{R}^{n \times n}}$ such that

$$\begin{equation*}{\rm{\ }}{\rho _{kj}} = \frac{{{\rm{E}}\left[ {\left( {{a_k} - {\mu _k}} \right)\left( {{a_j} - {\mu _j}} \right)} \right]}}{{{\sigma _k}{\sigma _j}}}\ \end{equation*}$$

where ${\mu _k}$, ${\sigma _k}$ and ${\mu _j}$, ${\sigma _j}$ are means and standard deviations for genes $k$ and $j$ across samples. Each sample (i.e. a column vector of $n$ measurements) is then linearly transformed (23) by the correlation matrix to reflect cross-sample dependencies as implemented by a matrix multiplication, ${A_1} = \ \rho \times A \in {\mathbb{R}^{n \times m}}$ which enables a computationally-efficient transformation.

#### Step 2. Discrete Fourier transformation

Each column of ${A_1}$, representing transformed molecular measurements of a cell, is then undergone discrete *Fourier transformation (DFT)*. We used the fast Fourier transform (FFT) (17), an efficient method for computing the DFT. For vectors $X$ and $Y$ of length $n$, DFT transformation, $Y = DFT( X )$ was defined as (24)

$$\begin{equation*}Y \left( k \right) = \mathop \sum \limits_{j = 1}^n X\left( j \right){e^{\frac{{ - 2\pi i}}{n}\left( {j - 1} \right)\left( {k - 1} \right)}} ,\quad k = 1,\ \ldots ,\ n\end{equation*}$$

where ${e^{2\pi i/n}}$ is a primitive *n*th root of 1. The magnitude (absolute value) of the fast Fourier transformed vector represents the *power per unit of the signal* which was then normalized by the number of variables $n$. We observed that taking the absolute value of the correlation-transformed measures ${A_1}$ enhances feature extraction performance (in reducing dataset complexity) after Fourier transformation. Therefore, the final matrix for Step 2 was computed as ${A_2} = \ | {{\rm{DFT}}( {| {{A_1}} |} )} |/n \in {\mathbb{R}^{n \times m}}$.

#### Step 3. Spectral entropy estimation

The spectral entropy (SE) of a signal is a measure of its spectral power distribution which treats the signal's normalized power distribution as a probability distribution and calculates the Shannon entropy (18) of it to describe the irregularity of energy/power distribution representing the complexity of a system (22). SE has been used for feature extraction in signal processing across diverse applications, e.g. (25). In SE estimation, the normalized PSD has been viewed as a Probability Density Function (integral is equal to 1) which is then used to estimate the information content.

Inspired by the spectral entropy estimation, the probability distribution of each sample/cell was estimated via scaling each feature by the sample's marginal sum and used to estimate the information content (or self-information as per Shannon's definition) of each variables, i.e. $I ( {k,j} ) = - {\log _2}P( {k,j} )$ where $P( {k,j} ) = {A_2}( {k,j} )/\mathop \sum \limits_{k = 1}^n {A_2}( {k,j} )$ for $k = 1,\ \ldots ,\ n$ and $j\ = \ 1,\ \ldots ,\ m$. The *entropy per unit of the spectrum* was then estimated as $H\ ( {k,j} ) = \ P( {k,j} )I( {k,j} )$. The final matrix for Step 3, ${A_3} \in {\mathbb{R}^{n \times m}}$, was then estimated by subtracting the entropy measure of each feature from its average entropy across samples to remove the effect of the ‘background’ information conveyed by that feature, i.e.

$$\begin{equation*} {A_3} ( {k,j} ) = \frac{1}{m}\mathop \sum \limits_{j = 1}^m H( {k,j} ) - H( {k,j} ). \end{equation*}$$

#### Step 4. Scaling between zero and one

Finally, the values of each sample represented in columns of ${A_3}$ is scaled so that its range is in the interval [0,1].

### Assessing the transformation dependency on the order of the features

It has been proven by Lanczos and Gellaithe (26) that Fourier analysis can be used to search for hidden periodicities in ‘random sequences’ wherein an exact value cannot be predicted for a future instant of the sequence. We, therefore, assumed that extracted patterns are independent of the initial random ordering of the genomic features. To assess this assumption quantitatively, we ran an experiment on a selected RNA-seq dataset wherein a gene ordering was randomly picked as the ‘reference order’ and then the order of transcripts was shuffled 100 times prior to scPSD transformation. After applying scPSD, the transformed matrices were rearranged to unify gene ordering based on the ‘reference order’. Accordingly, for each gene *k* in cell *j*, root mean square deviation (RMSD) was calculated estimating the deviation of the transformed gene expression compared to the corresponding transformed value in the reference matrix. Low RMSD values indicate that latent features extracted via scPSD transformation are not affected by the initial random ordering of the genomic measurements. Any initial random ordering of the features should remain the same across cells or samples.

### Internal validation measures

To quantify the compactness and separation of annotated cell-type clusters prior to any downstream analysis, we calculated internal validation measures (IVMs) for groups of cells defined by cell-type annotations provided with each published dataset. Two measures were used for this purpose: silhouette score (SS) and variance ratio criterion (VRC) as defined below:

*Variance ratio criterion*: VRC^21^ is the ratio of between-cluster dispersion to within-cluster dispersion and is defined as per equation below where *k* is the number of clusters, *n* is the number of data points, BGSS is the between group sum-of-squares, and WGSS is the within group sum-of-squares. Larger values of VRC indicate high dispersion between clusters and low dispersion within clusters.

$$\begin{equation*}{\rm{VRC }} = \frac{{BGSS}}{{k - 1}}\Big/\frac{{{\rm{WGSS}}}}{{n - k}} \end{equation*}$$

*Silhouette score*: SS is calculated as the mean silhouette coefficient over the dataset, and varies between −1 and 1 with larger values being better^20^. A high silhouette score indicates that each point is more similar to points in its own cluster than to points from other clusters. Assume that data have been clustered via any technique (or as per annotations) into *k* clusters (i.e. cell types). For each point $i$ in cluster ${C_i}$ (i.e. $i \in {C_i}$ assuming $| {{C_i}} | >1$), the silhouette coefficient is defined as:

$$\begin{equation*}{\rm{s }}\left( i \right) = \frac{{b\left( i \right) - a\left( i \right)}}{{\max \left( {b\left( i \right),a\left( i \right)} \right)}}\ \end{equation*}$$

where $a( i )$ is the average distance, $d( {i,j} )$, of point $i$ to each other point within the same cluster, ${C_i}$, and $b( i )$ is the average nearest-neighbor distance to each cluster formulated as:

$$\begin{equation*}a \left( i \right) = \frac{1}{{\left| {{C_i}} \right| - 1}}\ \mathop \sum \limits_{j \in {C_i},i \ne j} d\left( {i,j} \right),{\rm{\ }}b \left( i \right) = \mathop {\min }\limits_{k \ne i} \mathop {\frac{1}{{{C_k}}}\sum d\left( {i,j} \right)}\limits_{j \in {C_k}} \ \end{equation*}$$

Kendall's *W* (27) was calculated to measure the concordance in rankings of SSs of datasets as estimated by different distance measures (Euclidean, standardized Euclidean, cosine, and correlation). Kendall's *W* is calculated as follows:

$$\begin{equation*}W = \frac{{12\mathop \sum \nolimits_{i = 1}^n {{\left( {{R_i} - \bar{R}} \right)}^2}}}{{{m^2}\left( {{n^3} - n} \right)}}\ \end{equation*}$$

where ${R_i}$ is the sum of ranks for the *i*th dataset, $\bar{R}$ is the average *R* across all datasets, *n* is the number of variables (datasets), and *m* is the number of ‘judges’, i.e. distance measures. Permutation testing was used to estimate *p*-values.

### Multiclass Fisher's discriminant ratio

The Fisher's discriminant ratio, ${f_{ij}},$ was used as a separability measure of two classes of $i$ and $j$ and defined as (28)

$$\begin{equation*}{\rm{ }}{f_{ij}} = \frac{{{{\left( {{\mu _i} - {\mu _j}} \right)}^2}}}{{\sigma _i^2 + \sigma _j^2}}\ \end{equation*}$$

where ${\mu _i}$, $\sigma _i^2$ and ${\mu _j}$, $\sigma _j^2$ are means and variances for classes $i$ and $j$. Consider ${f_i} = \ ( {{f_{i1}},{f_{i2}},\ \ldots ,{f_{iN}}} )$ a vector representing the pairwise Fisher's discriminant ratio between class $i$ and $j$ for $j\ = \ 1 \ldots N$, where $n$ is the total number of classes (cell types). We defined ${F_i}$ as the approximate integral of ${f_i}$ estimated via the trapezoidal integration implemented by *trapz* function in MATLAB or R. Finally, the multi-class Fisher's discrimination ratio (mFDR) was calculated as

$$\begin{equation*}mFDR = \frac{1}{N} \mathop \sum \limits_{i = 1}^N {F_{\rm{i}}}\end{equation*}$$

### Normalization

As a pre-processing step prior to the scPSD transformation, multiple commonly used bulk RNA-seq normalization methods as well as single-cell-specific methods were used including:

*Trimmed means of M-values* (*TMM*) (29) which estimates the scaling factor based on the overall expression fold-change between the sample and a reference sample. The reference sample is the one which has an upper quartile closest to the mean upper quartile of all samples. TMM is implemented in the Bioconductor R package *edgeR*.

*Count per million* (*CPM*) (30) uses as the scaling factor the sum of the read counts across all transcripts in a sample multiplied by one million.

*Scone* (31) assesses the efficacy of various normalization workflows prior to finalizing their data normalization strategy. Scone is implemented in the Bioconductor R package *scone* (31). The default setting was chosen to select a top ranked method among scone library wrapper (31), upper-quartile scaling normalization (32), full-quantile normalization (33), and relative log-expression scaling normalization (34).

*Linnorm* (35) performs a prior logarithmic transformation on the expression data, and the dataset is fitted to a linear model that does not need to go through the origin. This allows expression level to be adjusted both linearly and exponentially. Bioconductor R package *linnorm* (35) were used with the default settings.

*Scran* (36) computes the scaling factors on pooled expression measures and then deconvolved to obtain cell-specific factors. The method is implemented in the Bioconductor R package *scran* (36). Pool sizes from 20 to the 100 (intervals of five) were considered.

*Seurat* (37) divides the transcript counts for each cell by the total counts for that cell and multiplied by the scale factor (i.e. default scaling factor is 10 000) followed by natural-log transformation. *Seurat* is implement in the Bioconductor R package *Seurat* (37).

*Signac* (38) is an extension of Seurat for the normalization and analysis of single-cell chromatin datasets. It computes term frequency-inverse document frequency (TF-IDF) normalization of the peak matrix by dividing the accessibility of each peak in a cell by the cell's total accessibility and multiplying this by the inverse accessibility of the peak in the cell population. This TF-IDF matrix is then log-transformed (37).

## RESULTS AND DISCUSSION

The scPSD feature transformation workflow is presented in Figure 1A. It can fit into any single-cell computational pipeline following upstream pre-processing (e.g. normalization, filtration, batch removal) to improve data quality and streamlining downstream computations. The transformation is independent of initial random ordering of genomic features (assuming the same random ordering of features across cells or samples) as demonstrated by low root mean square deviation (RMSD) of randomly shuffled features after transformation (2.47e−18 ≤ RMSD ≤ 4.33e−2, Supplementary Figure S1).

We assessed the performance of scPSD transformation, independent of any downstream analyses, in improving the cell-type clustering tendency and reducing the complexity of single-cell omics data. We previously proposed (14) a supervised application of internal validation measures (IVMs) such as silhouette score (SS) (39) and variance ratio criterion (VRC) (40), to quantify the compactness and separation of annotated cell-type clusters. We also defined a measure of the complexity of a multi-class dataset inspired by the Fisher's discriminant ratio (FDR) (28) (detailed in online Methods) to quantify the pairwise difference and dispersion of individual features among different cell types.

We comprehensively evaluated the effect of scPSD transformation in improving clustering tendency and complexity of single-cell transcriptomics data across 25 scRNA-seq datasets representing 14 tissue types, 10 sequencing protocols that resolved between 4 and 56 distinct cell-types (Supplementary Table S1). Upon selecting these datasets, we carefully assessed the underlying cell-type determination approaches (detailed in Supplementary Table S1) to incorporate trustworthy annotations for a reliable performance evaluation.

We have shown that scPSD significantly improves data quality (as measured by SS with Euclidean distance metric, VRC and our modified FDR) over these 25 RNA-seq datasets (Figure 1B and Supplementary Figures S2 and S3) while being efficient in time (Figure 1C). We applied scPSD on raw and normalized data. Multiple commonly-used normalization methods such as trimmed means of *M*-values (TMM) (29), count per million (CPM) (30), and Seurat (37) as well as single-cell-specific methods namely scone (31), Linnorm (35) and scran (36) were used as a pre-processing step prior to the transformation. We observed that while the type of normalization significantly affects data quality before transformation, after conducting scPSD, the quality of transformed data is not influenced by the normalization method, i.e. *P*-value of ANOVA test across different normalization methods on log-transformed VRC measures is significant before scPSD transformation (*P*= 3.57E−11), but insignificant afterwards (*P*= 0.478). This shows that scPSD transformation not only reduces the complexity of datasets for downstream analyses, but also can be used to harmonize single-cell omics data derived from diverse preprocessing pipelines for reuse, integration, and construction of cell atlases.

Furthermore, we assessed the effect of distance metrics on SSs by considering Euclidean, standardized Euclidean, one minus correlation, and one minus cosine similarity measures on a subset of scRNA-seq datasets (Supplementary Figure S4). We observed a high concordance between measures after transformation (Kendall's *W*  =  0.99, *P* < 10E−4) which indicates that cluster separability after scPSD transformation is independent of the distance measure. The effect of distance metrics on the separation of cell types was also assessed qualitatively for selected datasets. Accordingly, 2D t-SNE embeddings were estimated using different metrics of similarities and the corresponding scatter plots were visualized. Likewise, the visual separation among clusters was consistent after scPSD transformation regardless of the choice of the distance measure (Supplementary Figure S4). Beyond this analysis, all measures of SS in this study were estimated using the Euclidean distance.

Feature extraction is essential to improve the performance of machine learning algorithms. Supervised classification methods, for instance, have been widely adopted for automatic cell labelling to predict the identity of each cell by learning from an annotated training data (41). We compared the performance of the general-purpose support vector machine (SVM), the best performing classifier based on a former benchmarking study on scRNA-seq data (41), as well as other commonly used classifiers (i.e. random forest (RF) and *k*-nearest neighbor (KNN)) before and after scPSD feature extraction. For each dataset, the performance was evaluated based on the classification accuracy over a holdout test set (20% random split of a dataset) as well as the training computation time. The results clearly support a significant improvement in both metrics (due to the reduced complexity and faster convergence) irrespective of the choice of classifier or the upstream pre-processing approach (Figure 1D, E, and Supplementary Figure S5).

We further examined the effectiveness of scPSD transformation in facilitating the detection of rare cell populations. Rare or low abundant cell types within complex tissues can play important roles in normal development or disease progression (e.g. stem and progenitor cells, and circulating tumour cells) (42). Therefore, identifying rare cell populations can be of significant interest and the performance of rare cell type identification may not be consistent with the general classification performance. We reported the percentage of correctly classified cells belonging to the smallest cell-type population in each dataset, ranging from 2 to 500 cells, before and after scPSD transformation on raw and normalized datasets. We observed a clear improvement in identifying rare cells after transformation (Figure 1F and confusion matrices in Supplementary Figure S6).

We originally trained classifiers on the full set of genes. Classifiers, however, are often sensitive to the number of features (genes) used (41) necessitating a careful feature selection prior to classification. To assess the sensitivity of the classification performance to the number of features, we randomly selected 10% of genes from a modest-sized scRNA-seq dataset (deng reads (43)) and obtained SVM test accuracy before and after scPSD transformation. We then added another 10% of holdout genes, obtained the accuracy, and continued until accumulating all genes. This whole procedure was repeated 100 times to account for the random nature of the feature selection. Interestingly, we observed that the classifier has become extremely robust to feature elimination/selection upon scPSD transformation (Figure 1G).

Furthermore, to compare the effectiveness of scPSD feature extraction with feature extraction via dimensionality reduction, we studied 33 DR methods (Supplementary Table S4), extracted low-dimensional latent features across multiple datasets, and assessed the clustering tendency of cell types as measured by VRC and SS. We observed significantly higher IVMs using scPSD transformed features compared to features obtained by different DR methods (Supplementary Figure S7). Overall, scPSD can be used as a standalone feature extraction or precede a DR method to enable visualization (Supplementary Figure S3) or reduce data for more efficient downstream analyses.

Single-cell data is often compiled from multiple experiments with differences in handling personnel, capturing times, and technology platforms resulting technical variations or batch effects which can confound biological differences of interest (44). Feature extraction has the potential to reduce noise, redundancy, and irrelevant variations in data (45). Therefore, even though scPSD is not particularly developed to correct batch effects and can be preceded by a batch-removal algorithm as part of the upstream analyses, we presume that the cell-type separation would be relatively enhanced after scPSD transformation even at the presence of substantial batches. To assess this presumption, we employed four combined datasets (detailed in Supplementary Table S5) representing different batch-effect scenarios as suggested previously (44). These scenarios include (i) batches with identical cell-types and sequencing protocols but different capturing times, among others, (ii) batches with identical cell types but different protocols and (iii) batches containing non-identical cell types as well as different protocols. Overall, we observed notable improvement in clustering tendency of similar cells across batches after feature extraction by scPSD as measured by SS and VRC (Supplementary Table S5). Interestingly, 2D t-SNE plots show that identical cell-types from different batches are often combined or form near-by clusters after scPSD transformation (Supplementary Figure S8).

Beyond cell-type classification and clustering tendency, we assessed the utility of scPSD in improving developmental trajectory inference (TI). We studied 20 datasets representing diverse trajectory types, i.e. linear, bifurcation, multifurcation, and tree (Supplementary Table S6) and used minimum spanning tree (MST), a previously-shown (46) well-performing method, to infer topologies. As recommended by Saelens *et al* (46), we used multiple metrics for comparing trajectories including the Hamming–Ipsen–Mikhailov (HIM) (47) metric, F1 between branch assignments, and correlation between geodesic distances (46). Our initial results (Supplementary Figure S9) show that the MST average performance across all datasets measured by different metrics significantly improved after applying scPSD transformation (paired t-test *P*-value < 0.01) though the method performance was variable across datasets with different trajectory types (Supplementary Table S7).

**Figure 2.**
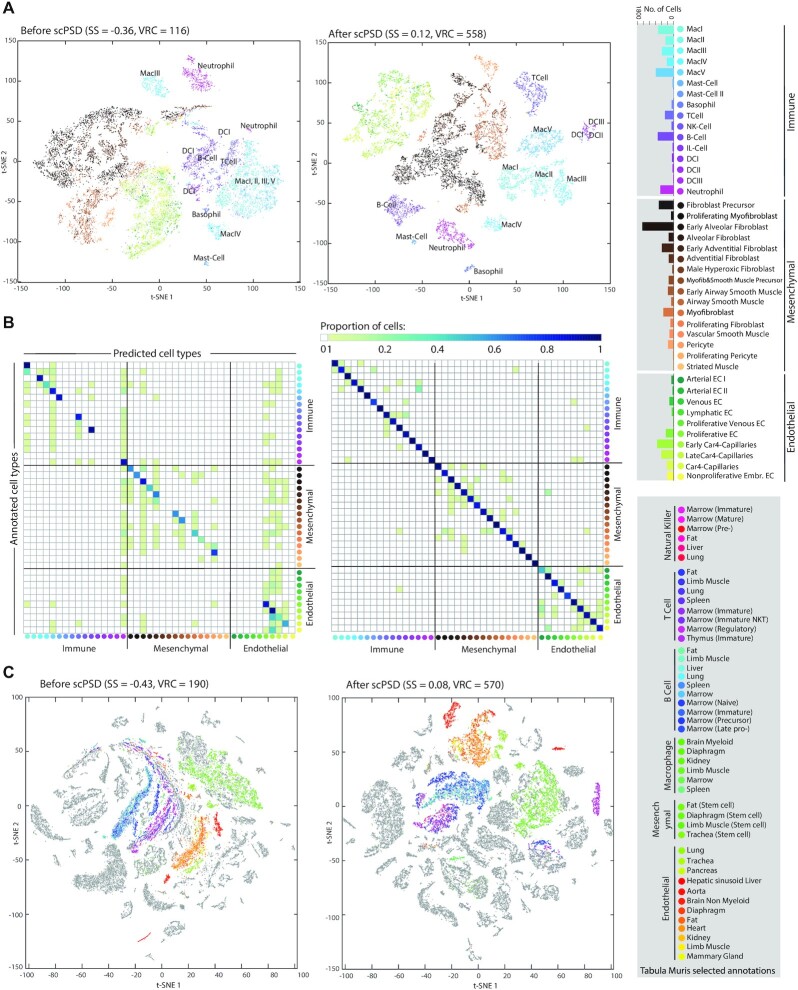
Evaluating scPSD performance on an atlas of the murine lung immune compartment and Tabula Muris mouse transcriptomic cell atlas. (**A**) t-SNE 2D visualizations (plus SS and VRC measures) before and after scPSD transformation of in-house scRNA-seq profiles capturing murine lung immune cell landscape combined with mesenchymal and endothelial cell subpopulations during postnatal development (data detailed in Supplementary Table S2). The combined profile was CPM normalized prior to visualization and transformation; immune cell subpopulations were annotated on the plots. (**B**) confusion matrices detailing the performance of SVM classification on test set (20% randomly held out cells) before and after scPSD transformation. Rows represent annotations (i.e. true classes) while columns represent predictions. Confusion matrices report the proportion of false positives, false negatives, true positives, and true negatives allowing more detailed analysis of cell-specific miss-classification. (**C**) t-SNE visualizations of Tabula Muris transcriptomics cell atlas accompanied with clustering tendency metrics (SS and VRC) for qualitative and quantitative evaluation of scPSD transformation on an entire organism. The immune, mesenchymal, and endothelial cells from different tissues were color-coded, other cells were grayed out however are explorable via interactive Supplementary Files 1 and 2. All t-SNE embeddings were generated using the ‘approximate’ method (i.e. KNN search) as implemented in MATLAB *tsne* function.

In addition, we assessed scPSD performance on three in-house, well-characterized single cell transcriptomics datasets (Supplementary Table S2) where scRNA-seq combined with fluorescent multiplexed in situ hybridization and flow cytometry were used to characterize changes in composition of immune cells (48) (5,234 cells and 16 cell subtypes), mesenchymal cells (49) (5479 cells and 16 subtypes), and endothelial cells (50) (2930 cells and 10 subtypes) in the murine lung during early postnatal development. Across three datasets, sequencing reads were obtained following the same protocol (Smart-Seq2) and bioinformatics pipeline resulting identical sequencing coverage. We applied scPSD on the CPM-normalized combined dataset (including 13 643 cells, 42 subtypes and 18 072 transcripts) and observed improvement in separation and dispersion of distinct cell-types (measured by SS and VRC) after scPSD feature extraction (Figure 2A). Strikingly, scPSD transformation disentangled the representation of the complex landscape of proliferative macrophages comprising multiple types of macrophages, dendritic cells, granulocytes, and lymphocytes as visualized by t-SNE 2D scatter plots before and after scPSD transformation (Figure 2A).

Confusion matrices in Figure 2B detail the cell-specific (mis-)classification rate on test set (20% holdout samples) using an SVM classifier trained on 80% of the combined dataset. Interestingly, while scPSD significantly improves classification performance (Supplementary Table S3), misclassified cells are often within the same cellular category in contrast to the pre-scPSD prediction where, for instance, multiple immune or mesenchymal cells were predicted as endothelial cells. Of note, since scPSD is an unsupervised procedure (i.e. does not rely on cell annotations), misclassified cells may also indicate occasional errors in the original annotations.

Beyond individual organs, we corroborated the efficacy of scPSD to process the single-cell transcriptomic data of an entire organism, the *Tabula Muris* mouse cell atlas (51), comprising over 100,000 cells from 20 organs and tissues. The scPSD transformation was efficient (84 seconds using 16 CPUs on UNSW HPC platform, Katana) and enhanced cell-type separation as measured by VRC and SS (Figure 2C). Interestingly, the t-SNE 2D plots show close proximities, yet often with distinct boundaries, in latent feature space among cell types that are shared between tissues, e.g. immune, mesenchymal, and endothelial cells from different anatomical locations (Figure 2C). To enable further visual investigation of relationships between cells from different organs, the interactive t-SNE plots of *Tabula Muris*, before and after scPSD transformation, were made available as Supplementary Files 1–2.

**Figure 3.**
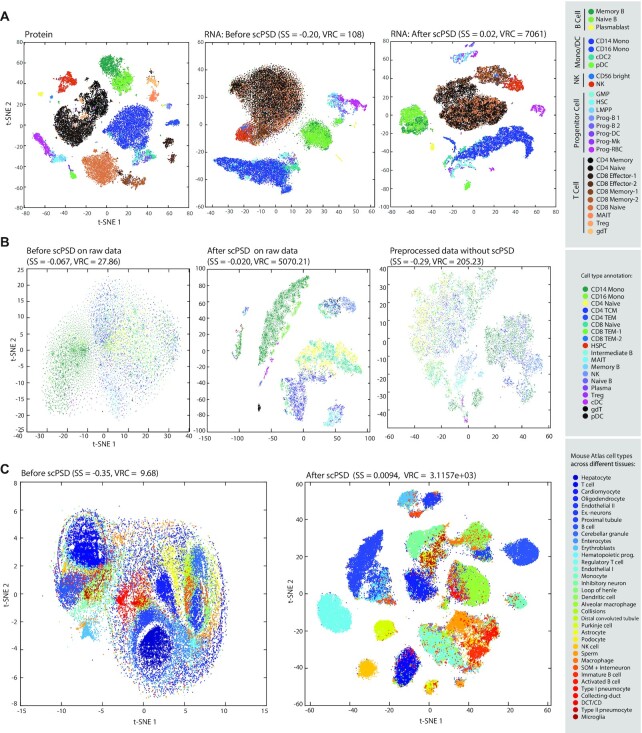
Performance validation on a CITE-seq dataset and evaluation of the scPSD applicability to scATAC-seq datasets. (**A**) t-SNE 2D plots of a CITE-seq dataset of bone marrow cells where immunophenotypes are measured in parallel with transcriptomes including the visualization of cell subpopulations based on cell-surface protein measurements (left plot), CPM-normalized scRNA-seq profiles before scPSD transformation (middle plot) and afterwards (right plot). (**B**) t-SNE visualizations of a 10X Genomics scATAC-seq dataset of human PBMC (downloaded from ‘filtered feature barcode matrix (HDF5))’ without pre-processing via normalization and feature selection (left plot), after scPSD transformation on non-processed data (middle plot) and after preprocessing including normalization and feature selection using ‘RunTFIDF’ and ‘FindTopFeatures’ (with q75 cutoff) functions implemented by signac’ library in R (right plot) (**C**) t-SNE visualizations of mouse single-cell atlas of chromatin accessibility before and after scPSD transformation; for both plots profiles are TFIDF normalized and features with zero values in more than 95% of cells were filtered out prior to visualization and transformation. The corresponding interactive plots are available as Supplementary Files 3–4. All t-SNE embeddings were generated using the ‘approximate’ method (i.e. KNN search) as implemented in MATLAB *tsne* function.

As another level of validation, we used a CITE-seq dataset (52) of bone marrow cells measuring transcriptomes in parallel with 25 cell-surface proteins representing well-characterized markers. The protein expression clearly discriminates immune subpopulations (Figure 3A) and can be considered as a gold standard for enumerating cell subsets based on quantitative differences in surface markers. While RNA-sequencing measures cannot differentiate cell-subtypes *a priori*, scPSD transformation enables high-resolution separation of cell types in concert with marker-based immunophenotyping (Figure 3A).

Beyond single cell transcriptomics, the scPSD transformation is expected to be applicable to other single-cell omics modalities as well as bulk sequencing data. As a proof of principle, we applied scPSD on two scATAC-seq datasets to assess its performance on single-cell measurements of chromatin accessibility. First, we analyzed the 10× Genomics scATAC-seq dataset of human PBMC granulocytes comprising 108 377 peaks across 11 909 cells. Figure 3B shows the t-SNE visualizations of the raw profile, preprocessed data (TFIDF normalization followed by inclusion of top 25% most common features using *signac* library in R (38)), and raw data after scPSD transformation. Together, the results demonstrate that although pre-processing enhances the separation of cell subpopulations, scPSD transformation further improves cell subtype clustering both visually and quantitatively (as measured by SS and VRC, Figure 3B). We further applied scPSD on the single-cell atlas of chromatin accessibility in mouse capturing ∼100 000 cells across 13 different tissues and similarly observed that the scPSD transformation significantly improves cell-type separation quantitatively (measured by SS and VRC) and qualitatively (shown by t-SNE plots), cf., Figure 3C, and Supplementary Files 3–4 for interactive plots.

Overall, we substantively demonstrated that the scPSD feature extraction reduces the complexity of single-cell sequencing data. However, it is important to note that scPSD transforms variables into a *latent feature space* wherein the features are not directly equivalent to the features in the original space. Therefore, transformed features are best suited for cell- or sample-level downstream analyses such as cell-type classification where such latent features can be used as predictors of machine learning models. However, the latent features cannot be directly used in feature-level analyses such as differential expression analyses. The extension of the scPSD method to enable feature-level analyses is a future direction.

## DATA AVAILABILITY

The scPSD method has been implemented in MATLAB (https://github.com/VafaeeLab/psdMAT), Python (https://github.com/VafaeeLab/psdPy) and R package (https://github.com/VafaeeLab/psdR). To assure the reproducibility of the reported results, the data and pipeline developed for this study are available at (https://github.com/VafaeeLab/psdMAT).

## Supplementary Material

gkac436_Supplemental_File
