# Supplementary material for: Disentangling single-cell omics representation with a power spectral density-based feature extraction
Source: Nucleic Acids Res. 2022 May 25;50(10):5482–92. doi: 10.1093/nar/gkac436 (PMC9178020; doi:10.1093/nar/gkac436)
Supplement: gkac436_Supplemental_File [file gkac436_supplemental_file.pdf]

Supplementary Information

Disentangling single-cell omics representation with a power spectral density-based feature extraction

Seid Miad Zandavi[1], Forrest C Koch[1], Abhishek Vijayan[1], Fabio Zanini[2,3], Fatima Valdes Mora[4,7], David Gallego Ortega[5], Fatemeh Vafaee[1,3,6]*

[1] School of Biotechnology and Biomolecular Sciences, University of New South Wales (UNSW Sydney), Australia
[2] Prince of Wales Clinical School, UNSW Sydney, Australia
[3] Cellular Genomics Future Institute, UNSW Sydney, Australia
[4] Children's Cancer Institute, Lowy Cancer Research Centre, UNSW Sydney, Australia
[5] School of Biomedical Engineering, University of Technology Sydney (UTS), Australia
[6] UNSW Data Science Hub (uDASH), UNSW Sydney, Australia
[7] School of Women's and Children's Health, Faculty of Medicine, UNSW, Sydney, Australia

*** Correspondence to: f.vafaee@unsw.edu.au**

Supplementary Tables

Supplementary Table 1. Summary of scRNA-seq datasets used for the evaluation of scPSD.

Supplementary Table 2. Summary of scRNA-seq in-house datasets.

Supplementary Table 3. Cell-specific SVM classification performance.

Supplementary Table 4. Summary of dimensionality reduction methods

Supplementary Table 5. Summary of combined scRNA-seq datasets with defined batch effects.

Supplementary Table 6. Summary of trajectory inference (TI) datasets

Supplementary Table 7. Details of metrics comparing the performance of MST trajectory inference before and after scPSD transformation.

Supplementary Figures

Supplementary Fig 1. Independence of transformation on the initial random ordering.

Supplementary Fig 2. Variance Ratio Criterion (VRC), Silhouette Score (SS) and multiclass Fisher's Discriminant Ratio (mFDR) across scRNA-seq datasets.

Supplementary Fig 3. 2D-visualisation (t-SNE dimensionality reduction) of scRNA-seq datasets.

Supplementary Fig 4. Effect of distance metrics on quantitative and qualitative separation of cell types before and after scPSD transformation.

Supplementary Fig 5. Heatmaps representing cell type classification performance across each dataset before and after scPSD transformation.

Supplementary Fig 6. Confusion matrices (CMs) detailing the performance of SVM classification on test data (randomly picked 20% of dataset).

Supplementary Fig 7. (a) Heatmaps of the SS with Euclidean distance and VRC for 33 dimensionality reduction methods.

Supplementary Fig 8. The 2D t-SNE visualisation of datasets with defined batch effects.

Supplementary Fig 9. Trajectory inference (TI) analysis.

Supplementary Files

Supplementary Files 1 and **2**. Interactive MATLAB figures to investigate the 2D visualization of the Tabula Muris transcriptomics cell atlas before and after scPSD transformation, respectively; accessible at https://github.com/VafaeeLab/psdMAT

Supplementary Files 3 and **4**. Interactive MATLAB figures to investigate the 2D visualization of the chromatin accessibility cell atlas before and after scPSD transformation, respectively; accessible at https://github.com/VafaeeLab/psdMAT

Supplementary Tables

Supplementary Table 1. *Summary of scRNA-seq datasets used for the evaluation of scPSD.*

#	Dataset	Protocol	Accession	Species	Tissue type	Cells	Genes	Cell Types	Min cell type %	Max cell type %	UMI/ Read	Cell type determination*
1	chuBatch1 (1)	SMARTer	GSE75748	human	stem cells	350	19097	5	14	24.86	Read	Gold
2	chuBatch2 (1)	SMARTer	GSE75748	human	stem cells	425	19097	6	9.65	20.47	Read	Gold
3	Linnarsson (2) (cerebellum)	Chromium	SRP135960	mouse	brain	12312	17984	8	0.48	64.75	UMI	Prediction
4	Colon FACS (3)	Smart-Seq2	GSE132042	mouse	colon	4149	23433	6	0.6	19.62	Read	Silver
5	Fat FACS (3)	Smart-Seq2	GSE132042	mouse	fat	5862	23433	11	0.46	3.77	Read	Silver
6	Heart 10X (3)	Chromium	GSE132042	mouse	heart	654	23433	7	3.21	33.94	UMI	Prediction
7	Heart FACS (3)	Smart-Seq2	GSE132042	mouse	heart	7115	23433	11	0.16	17.95	Read	Prediction
8	Kidney FACS (3)	Smart-Seq2	GSE132042	mouse	kidney	865	23433	7	4.39	7.51	Read	Prediction
9	Liver FACS (3)	Smart-Seq2	GSE132042	mouse	liver	981	23433	6	2.96	19.98	Read	Prediction
10	Lung FACS (3)	Smart-Seq2	GSE132042	mouse	lung	1923	23433	17	0.1	0.68	Read	Silver
11	Pancreas FACS (3)	Smart-Seq2	GSE132042	mouse	pancreas	1961	23433	10	1.48	20.96	Read	Prediction
12	baron mouse (4)	inDrop	GSE84133	mouse	pancreas	1886	14878	13	0.32	11.56	UMI	Prediction
13	deng reads (5)	Smart-Seq2	GSE45719	mouse	embryo	268	22431	6	4.48	13.81	Read	Gold
14	fan (6)	SUPeR-seq	GSE53386	mouse	embryo	66	26357	6	10.61	33.33	Read	Gold
15	goolam (7)	Smart-Seq2	E-MTAB-3321	mouse	embryo	124	41480	5	4.84	25.81	Read	Gold
16	klein (8)	inDrop	GSE65525	mouse	embryo stem cells	2717	24175	4	11.15	34.34	UMI	Gold
17	li (9)	SMARTer	GSE81861	human	colorectal tumor	561	55186	9	4.1	12.3	Read	Prediction
18	manno human (10)	STRT-Seq UMI	GSE76381	human	brain	4029	20560	56	0.12	0.45	UMI	Prediction
19	manno mouse (10)	STRT-Seq UMI	GSE76381	mouse	brain	2150	24378	32	0.56	1.26	UMI	Prediction
20	marques (11)	C1	GSE75330	mouse	brain	5053	23556	13	1.5	9.12	Read	Prediction
21	pollen (12)	SMARTer	SRP041736	human	neonatal-foreskin	301	23730	11	2.33	17.94	Read	Prediction
22	tasic reads (13)	SMARTer	GSE71585	mouse	brain	1679	24150	18	0.54	16.38	Read	Silver
23	yan (14)	Tang	GSE36552	human	embryo	90	20214	6	6.67	17.78	Read	Gold
24	zhengmix4eq (15)	GemCode	SRP073767	human	immune	3994	15568	4	3.05	8.55	UMI	Gold
25	zhengmix4uneq (15)	GemCode	SRP073767	human	immune	6498	16443	4	7.69	46.14	UMI	Gold

*** Details of cell type assignment evaluation:**

Cell annotations were categorised as *gold standard* when there was prior information on cell types to be analysed, this includes the use of a canonical markers to FACS sort a specific cell population before performing the single-cell RNA-seq method, but also instances where the cell population was generated under controlled conditions (e.g. in vitro culture). Similarly, *silver standard* denotes some degree of previous knowledge of the biological system, including a high hierarchical level of cell enrichment (e.g., a cell lineage) prior single-cell RNA-seq analysis. Finally, *prediction* was assigned to the studies that did not include a priori information on the cell composition of the biological system or experiments that did not include a cell selection strategy and thus, cell annotation was performed through dimensionality reduction followed by clustering based gene expression profiles, cluster-specific markers were subsequently used to annotate the cell composition of the biological system.

Supplementary Table 2. *Summary of scRNA-seq in-house datasets used for the evaluation of scPSD.*

Dataset	Accession	Tissue type	Cells	Genes	# of Cell Types	Cell Types (#)
Domingo-Gonzalez and Zanini et al. (16)	GSE147668	mouse murine lung **immune** cells	5234	18072	16	Mac I (737), Mac II (391), Mac III (563), Mac IV (332), Mac V (847), mastCell (67), mastCell II (22), basophil (102), TCell (418), NKCell (111), BCell (755), ILCell (60), DC I (94), DC II (67), DC III (36), neutrophil (632).
Zanini et al. (17)	GSE172251	mouse murine lung **mesenchymal** cells	5479	18072	16	Fibroblast Precursor (764), Proliferating Myofibroblast (118), Early Alveolar Fibroblast (1618), Alveolar Fibroblast (250), Early Adventitial Fibroblast (598), Adventitial Fibroblast (241), Male Hyperoxic Fibroblast (55), Myofibroblast And Smooth Muscle Precursor (241), Early Airway Smooth Muscle (285), Airway Smooth Muscle (113), Myofibroblast (520), Proliferating Fibroblast (157), Vascular Smooth Muscle (201), Pericyte (281), Proliferating Pericyte (19), Striated Muscle (18)
Zanini et al. (18)	GSE159804	mouse murine lung **endothelial** cells	2930	18072	10	Arterial EC I (96), Arterial EC II (42), Venous EC (203), Lymphatic EC (82), Proliferative Venous EC (10), Proliferative EC (390), Early Car4 Capillaries (840), Late Car 4 Capillaries (622), Car 4 Capillaries (332), Nonproliferative Embryonic EC (313)

Supplementary Table 3. *Cell-specific SVM classification performance – sensitivity (**Sen**) and specificity (**Spe**), F1 score (**F1**), positive predictive value (**PPV**), and negative predictive value (**NPV**) over test set, i.e., 20% random holdout cells – using datasets detailed in Supplementary Table 2, before and after scPSD feature extraction (CPM normalization).*

	**Cell type**	**Cells* (Test)**	**Before scPSD**					**After scPSD**				
			**Sen**	**Spe**	**PPV**	**NPV**	**F1**	**Sen**	**Spe**	**PPV**	**NPV**	**F1**
**Immune**	Mac I	121	0.979	0.976	0.687	0.999	0.807	0.95	0.997	0.944	0.997	0.947
	Mac II	68	0	1	0	0.971	0	0.848	0.997	0.893	0.996	0.87
	Mac III	330	0.915	0.998	0.956	0.996	0.935	1	0.998	0.959	1	0.979
	Mac IV	128	0.418	0.997	0.778	0.986	0.544	0.896	1	1	0.997	0.945
	Mac V	137	0.848	0.965	0.625	0.989	0.719	0.966	0.998	0.977	0.998	0.972
	mastCell	80	0.882	1	1	0.999	0.938	1	1	1	1	1
	mastCell II	177	0	1	0	0.997	0	0.75	1	0.857	0.999	0.8
	basophil	141	0	1	0	0.992	0	1	1	1	1	1
	TCell	16	0.825	0.992	0.759	0.995	0.79	0.95	0.998	0.938	0.999	0.944
	NKCell	32	0	1	0	0.992	0	0.905	1	0.95	0.999	0.927
	BCell	154	0.942	0.999	0.977	0.997	0.959	0.985	0.998	0.964	0.999	0.975
	ILCell	79	0	1	0	0.995	0	0.929	0.999	0.813	1	0.867
	DC I	53	0	1	0	0.994	0	1	1	1	1	1
	DC II	56	0	1	0	0.996	0	0.917	0.999	0.733	1	0.815
	DC III	148	0	1	0	0.999	0	1	1	1	1	1
	neutrophil	24	0.898	0.937	0.412	0.995	0.565	0.977	1	1	0.999	0.988
**Mesenchymal**	Fibroblast Precursor	118	0.604	0.99	0.788	0.977	0.684	0.968	0.998	0.961	0.998	0.964
	Proliferating Myofibroblast	124	0.6	1	1	0.996	0.75	0.88	0.999	0.88	0.999	0.88
	Early Alveolar Fibroblast	38	0.861	0.928	0.621	0.98	0.722	0.967	0.994	0.958	0.995	0.962
	Alveolar Fibroblast	114	0.477	1	0.955	0.992	0.636	0.955	0.999	0.955	0.999	0.955
	Early Adventitial Fibroblast	70	0.636	0.98	0.597	0.983	0.616	0.926	0.996	0.918	0.997	0.922
	Adventitial Fibroblast	16	0	1	0	0.981	0	0.887	0.999	0.94	0.998	0.913
	Male Hyperoxic Fibroblast	67	0	1	0	0.995	0	0.846	0.999	0.846	0.999	0.846
	Myofibroblast & Smooth Muscle Precursor	67	0.607	0.999	0.895	0.992	0.723	0.911	0.999	0.927	0.998	0.919
	Early Airway Smooth Muscle	77	0.603	0.997	0.82	0.99	0.695	0.956	0.997	0.878	0.999	0.916
	Airway Smooth Muscle	21	0	1	0	0.991	0	1	1	1	1	1
	Myofibroblast	23	0.468	0.99	0.691	0.975	0.558	0.863	0.997	0.93	0.994	0.895
	Proliferating Fibroblast	19	0.217	0.999	0.714	0.993	0.333	0.87	0.999	0.87	0.999	0.87
	Vascular Smooth Muscle	21	0.026	1	1	0.986	0.051	0.921	0.999	0.946	0.999	0.933
	Pericyte	44	0.754	0.994	0.741	0.995	0.748	0.983	0.999	0.966	1	0.974
	Proliferating Pericyte	13	0	1	0	0.999	0	1	1	1	1	1
	Striated Muscle	17	0	1	0	0.999	0	1	1	1	1	1
**Endothelial**	Arterial EC I	2	0	1	0	0.994	0	0.5	0.998	0.615	0.997	0.552
	Arterial EC II	57	0	1	0	0.998	0	1	0.999	0.667	1	0.8
	Venous EC	14	0	1	0	0.988	0	0.844	0.999	0.871	0.998	0.857
	Lymphatic EC	8	0	1	0	0.993	0	0.947	1	0.947	1	0.947
	Proliferative Venous EC	4	0	1	0	0.999	0	0.75	1	1	1	0.857
	Proliferative EC	25	0.87	0.986	0.644	0.996	0.74	0.961	0.997	0.914	0.999	0.937
	Early Car4 Capillaries	12	0.946	0.897	0.346	0.997	0.506	0.912	0.993	0.882	0.995	0.897
	Late Car 4 Capillaries	6	0.307	0.979	0.393	0.97	0.345	0.921	0.998	0.946	0.997	0.933
	Car 4 Capillaries	3	0.552	0.996	0.771	0.989	0.644	0.94	0.999	0.955	0.999	0.947
	Nonproliferative Embryonic EC	4	0	1	0	0.974	0	0.9	0.997	0.875	0.997	0.887

***** Number of each cell-type in the holdout test set; total number of test cells are 2,728 that is equivalent to 20% of all cells across three populations of immune (5234), mesenchymal (5479), and endothelial (2930) cells.

Supplementary Table 4. *Summary of dimensionality reduction methods studied in Supplementary Figure 6.*

Method	Abbrev.	Brief Description	Package
Bayesian decomposition (19)	BD	Uses a Gibbs sampler to estimate the posterior density of NMF factors	nimfa
Factor Analysis (20)	FA	Linear generative model with Gaussian latent variables	sklearn
Fast Independent Component Analysis (21)	FICA	Extracts statistically independent non-Gaussian signals	sklearn
Gaussian Random Projection (22)	GRP	Projecting the original input space on a randomly generated matrix where components are drawn from a normal distribution	sklearn
Incremental PCA (23)	IPCA	A mini-batch implementation of PCA	sklearn
Isometric Mapping (24)	ISOMAP	An extension on MDS that attempts to preserve geodesic distances	sklearn
Iterated Conditional Modes (19)	ICM	Like BD, but uses modes in place of random samples in the Gibbs sampler	nimfa
ivis (25)	IVIS	Uses Siamese neural networks to embed data	ivis
Kernel PCA (26) (with cosine, polynomial, and radial basis function transformations)	KPCA-(COS/ POL/RBF)	Utilizes the kernel trick to allow for non-linear representations of the data.	sklearn
Latent Dirichlet allocation (27)	LDA	Bayesian generative model utilizing a Dirichlet prior over latent space	sklearn
Least squares NMF (28)	LSNMF	Alternative to multiplicative NMF which claims to have faster convergence	nimfa
Locally linear embedding(29)	LLE	Similar to isomap, but attempts to preserve local distances	sklearn
Non-negative Matrix Factorization(30,31)	NMF-(NNSVD/ LEE)	Factorizes a (non-negative) data into two non-negative matrices.	sklearn/ nimfa
Non-smooth NMF (nsnmf) (32)	NSNMF	Applies smoothing kernel into the factorization step reducing sparsity	nimfa
Potential of Heat-diffusion for Affinity-based Trajectory Embedding (33)	PHATE	Utilizes a heat diffusion model to preserve branching structures in data	phate
Principle Component Analysis (34)	PCA	Construct a low dimensional, orthogonal basis that maximally explains observed variance in the data.	sklearn
Probabilistic non-negative Matrix Factorization (35)	PMF	Assumption that data follows a multinomial distribution and performs expectation maximization	nimfa
Probabilistic sparse MF (36)	PSMF	Assumption of Gaussian noise within the data, and sparsity constraint added for factorization	nimfa
Sparse Autoencoder for Clustering, Imputing, and Embedding (37)	SAUCIE	Sparse Autoencoder Neural Network	saucie
Sparse nmf (38)	SNMF	Addition of sparsity constraint to factorization process	nimfa
sparse PCA (39)	SPCA(-BATCH)	An extension of PCA which aims to produce sparse (with many zeros) components	sklearn
Sparse Random Projection (40)	SRP	Similar to GRP, but matrix elements are drawn from a zero-inflated, centered uniform distribution.	sklearn
Laplacian eigenmaps (41)	SPECTRAL	Attempts to reconstruct the manifold in low dimensional space by preserving the distances of the nearest neighbors to each point	sklearn
t-distributed Stochastic Neighbour Embedding (42)	TSNE	Minimizes the KL divergence of the estimated distribution of points in high dimensional points with a similar low-dimensional distribution.	Mutlicore TSNE
Truncated Singular Value Decomposition (34)	TSVD	PCA without centring and scaling	sklearn
Uniform Manifold Approximation and Projection (43)	UMAP	Constructs a fuzzy topological representation of the data to preserve in the low dimensional embedding	Umap-learn
Variational autoencoder (44)	VASC	Uses neural network to map latent variables to zero-inflated negative binomial distribution	vasc
Variational projection (45)	VPAC	Uses a variational projection algorithm to infer a Gaussian mixture in latent space	vpac
Zero-inflated Factor Analysis (46)	ZIFA	Uses a latent variable model, similar to FA, in which is modulated by the addition of a zero-inflated layer meant to simulate dropout.	ZIFA

Supplementary Table 5. *Summary of combined scRNA-seq datasets with defined batch effects. Datasets were formed by combining scRNA-seq experiments on similar or identical tissues from a same organism (human or mouse). Each dataset comprises 2 batches which represent same set of genes, so datasets were simply integrated by features*.

**No**	**Experiment Name (#)** *(c.f. Supplementary Table 1)**	**Description**	**Technology**	**Batch Effects**	**Cell-type separation (VRC, SS)**	
					Before scPSD	After scPSD
**1**	chuBatch1 (# 1)<br>chuBatch2 (# 2)	Human Stem Cell	SMARTer	Same technology,<br>Same tissue	(86.2, 0.060)	(769.6, 0.680)
**2**	zhengmix4eq (# 24)<br>zhengmix4uneq (# 25)	Human PBMC	GemCode	Same technology,<br>Same tissue	(962.2, 0.116)	(20057.1, 0.6178)
**3**	TM Heart 10X (# 6)<br>TM Heart FACS (# 7)	Mouse Heart	Chromium<br>Smart-Seq2	Different technology,<br>Same tissue	(163.8, -0.273)	(2667.4, 0.585)
**4**	fan (# 14)<br>klein (# 16)	Mouse Embryo<br>(Stem Cell)	SUPeR-seq<br>inDrop	Different technology,<br>Different tissue	(49.6, 0.034)	(671.2, 0.267)

** Name and # of scRNA-seq experiments are based on Supplementary Table 1 which can be referred to get information.*

Supplementary Table 6. *Summary of trajectory inference (TI) datasets used for the evaluation of scPSD.* The R package suite created alongside the TI benchmarking study by Saelens et al(47) (https://github.com/dynverse) was used for performing the trajectory inference. Datasets uploaded to zenodo (https://zenodo.org/record/1443566) as part of the same study was used here; datasets were classified by Saelens *et al* as 'gold standard' if the reference trajectory was not extracted from the expression data itself, such as via cellular sorting or cell mixing or as 'silver standard', otherwise. Please refer to the original paper for further details on datasets.

No	Dataset	Accession	Gold/Silver	Trajectory type	# of Cells	# of Genes	Protocol
1	**aging-hsc-old_kowalczyk**	GSE59114	gold	linear	873	2815	Smart-Seq
2	**developing-dendritic-cells_schlitzer**	GSE60781	gold	linear	238	4480	fluidigm c1
3	**human-embryos_petropoulos**	E-MTAB-3929	gold	linear	1289	8772	fluidigm c1
4	**myoblast-differentiation_trapnell**	GSE52529	gold	linear	290	8772	fluidigm c1
5	**pancreatic-alpha-cell-maturation_zhang**	GSE87375	gold	linear	322	6138	smart-seq2
6	**neonatal-rib-cartilage_mca**	https://figshare.com/s/865e694ad06d5857db4b	silver	bifurcation	2221	2449	Microwell-Seq
7	**thymus-t-cell-differentiation_mca**	https://figshare.com/s/865e694ad06d5857db4b	silver	bifurcation	1607	2642	Microwell-Seq
8	**distal-lung-epithelium_treutlein**	GSE52583	silver	bifurcation	59	3491	fluidigm c1
9	**fibroblast-reprogramming_treutlein**	GSE67310	silver	bifurcation	355	3379	fluidigm c1
10	**hepatoblast-differentiation_yang**	GSE90047	silver	bifurcation	504	6138	smart-seq2
11	**macrophage-salmonella_saliba**	GSE79363	gold	multifurcation	60	6558	smart-seq2
12	**NKT-differentiation_engel**	GSE74596	gold	multifurcation	197	6799	fluidigm c1
13	**fetal-liver-fetal-hematopoiesis_mca**	https://figshare.com/s/865e694ad06d5857db4b	silver	multifurcation	2559	2470	Microwell-Seq
14	**placenta-trophoblast-differentiation_mca**	https://figshare.com/s/865e694ad06d5857db4b	silver	multifurcation	1001	2588	Microwell-Seq
15	**trophoblast-stem-cell-trophoblast-differentiation_mca**	https://figshare.com/s/865e694ad06d5857db4b	silver	multifurcation	19467	3157	Microwell-Seq
16	**mesoderm-development_loh**	GSE85066	gold	tree	504	8772	fluidigm c1
17	**embronic-mesenchyme-neuron-differentiation_mca**	https://figshare.com/s/865e694ad06d5857db4b	silver	tree	481	2969	Microwell-Seq
18	**epiblast-monkey_nakamura**	GSE74767	silver	tree	182	4320	SC3-seq
19	**hematopoiesis-clusters_olsson**	GSE70240, GSE70244, GSE70236	silver	tree	376	3594	fluidigm c1
20	**planaria-full_plass**	GSE103633	silver	tree	18837	4210	drop-seq

Supplementary Table 7. *Details of metrics comparing the performance of MST trajectory inference before and after scPSD transformation*. * Last column represents the overall score computed as the geometric mean of the other three metrics.

		**Before scPSD**				**After scPSD**			
**No**	Dataset	Correlation	$F1_{branches}$	HIM	Overall*	Correlation	$F1_{branches}$	HIM	Overall*
**1**	**aging-hsc-old_kowalczyk**	0.469	0.269	0.39	0.366	0.561	1	1	0.825
**2**	**developing-dendritic-cells_schlitzer**	0.737	0.378	0.608	0.553	0.627	1	1	0.856
**3**	**human-embryos_petropoulos**	0.799	0.456	0.733	0.644	0.741	1	1	0.905
**4**	**myoblast-differentiation_trapnell**	0.121	0.26	0.426	0.238	0.129	0.267	0.463	0.251
**5**	**pancreatic-alpha-cell-maturation_zhang**	0.43	1	1	0.755	0.491	1	1	0.789
**6**	**neonatal-rib-cartilage_mca**	0.129	0.281	0.677	0.291	0.452	0.563	0.885	0.608
**7**	**thymus-t-cell-differentiation_mca**	0.193	0.37	0.598	0.35	0.668	0.575	1	0.727
**8**	**distal-lung-epithelium_treutlein**	0.633	0.638	0.575	0.615	0.784	0.629	1	0.79
**9**	**fibroblast-reprogramming_treutlein**	0.704	0.531	0.685	0.635	0.598	0.447	0.519	0.518
**10**	**hepatoblast-differentiation_yang**	0.379	0.355	0.588	0.429	0.518	0.384	0.711	0.521
**11**	**macrophage-salmonella_saliba**	0.107	0.405	0.546	0.287	0.086	0.462	0.684	0.301
**12**	**NKT-differentiation_engel**	0.481	0.322	0.686	0.473	0.624	0.618	0.907	0.705
**13**	**fetal-liver-fetal-hematopoiesis_mca**	0.657	0.4	0.418	0.479	0.707	0.401	0.427	0.495
**14**	**placenta-trophoblast-differentiation_mca**	0.279	0.251	0.843	0.389	0.737	0.449	0.645	0.598
**15**	**trophoblast-stem-cell-trophoblast-differentiation_mca**	0.266	0.26	0.578	0.342	0.556	0.259	0.7	0.465
**16**	**mesoderm-development_loh**	0.542	0.356	0.449	0.443	0.593	0.379	0.643	0.525
**17**	**embronic-mesenchyme-neuron-differentiation_mca**	0.079	0.171	0.555	0.196	0.168	0.286	0.387	0.265
**18**	**epiblast-monkey_nakamura**	0.327	0.607	0.531	0.472	0.267	0.622	0.578	0.458
**19**	**hematopoiesis-clusters_olsson**	0.587	0.234	0.34	0.36	0.762	0.234	0.34	0.393
**20**	**planaria-full_plass**	0.267	0.108	0.436	0.233	0.466	0.129	0.555	0.322

Reference

1. Chu, L.-F., Leng, N., Zhang, J., Hou, Z., Mamott, D., Vereide, D.T., Choi, J., Kendziorski, C., Stewart, R. and Thomson, J.A.J.G.b. (2016) Single-cell RNA-seq reveals novel regulators of human embryonic stem cell differentiation to definitive endoderm. *Genome biology*, **17**, 1-20.
2. Zeisel, A., Hochgerner, H., Lönnerberg, P., Johnsson, A., Memic, F., Van Der Zwan, J., Häring, M., Braun, E., Borm, L.E. and La Manno, G.J.C. (2018) Molecular architecture of the mouse nervous system. *Cell*, **174**, 999-1014. e1022.
3. Schaum, N., Karkanias, J., Neff, N.F., May, A.P., Quake, S.R., Wyss-Coray, T., Darmanis, S., Batson, J., Botvinnik, O. and Chen, M.B.J.N. (2018) Single-cell transcriptomics of 20 mouse organs creates a Tabula Muris: The Tabula Muris Consortium. *Nature*, **562**, 367.
4. Baron, M., Veres, A., Wolock, S.L., Faust, A.L., Gaujoux, R., Vetere, A., Ryu, J.H., Wagner, B.K., Shen-Orr, S.S. and Klein, A.M.J.C.s. (2016) A single-cell transcriptomic map of the human and mouse pancreas reveals inter- and intra-cell population structure. *Cell systems*, **3**, 346-360. e344.
5. Deng, Q., Ramsköld, D., Reinius, B. and Sandberg, R.J.S. (2014) Single-cell RNA-seq reveals dynamic, random monoallelic gene expression in mammalian cells. *Science*, **343**, 193-196.
6. Fan, X., Zhang, X., Wu, X., Guo, H., Hu, Y., Tang, F. and Huang, Y.J.G.b. (2015) Single-cell RNA-seq transcriptome analysis of linear and circular RNAs in mouse preimplantation embryos. *Genome biology*, **16**, 1-17.
7. Goolam, M., Scialdone, A., Graham, S.J., Macaulay, I.C., Jedrusik, A., Hupalowska, A., Voet, T., Marioni, J.C. and Zernicka-Goetz, M.J.C. (2016) Heterogeneity in Oct4 and Sox2 targets biases cell fate in 4-cell mouse embryos. *Cell*, **165**, 61-74.
8. Klein, A.M., Mazutis, L., Akartuna, I., Tallapragada, N., Veres, A., Li, V., Peshkin, L., Weitz, D.A. and Kirschner, M.W.J.C. (2015) Droplet barcoding for single-cell transcriptomics applied to embryonic stem cells. *Cell*, **161**, 1187-1201.
9. Li, H., Courtois, E.T., Sengupta, D., Tan, Y., Chen, K.H., Goh, J.J.L., Kong, S.L., Chua, C., Hon, L.K. and Tan, W.S.J.N.g. (2017) Reference component analysis of single-cell transcriptomes elucidates cellular heterogeneity in human colorectal tumors. *Nature genetics*, **49**, 708-718.
10. La Manno, G., Gyllborg, D., Codeluppi, S., Nishimura, K., Salto, C., Zeisel, A., Borm, L.E., Stott, S.R., Toledo, E.M. and Villaescusa, J.C.J.C. (2016) Molecular diversity of midbrain development in mouse, human, and stem cells. *Cell*, **167**, 566-580. e519.
11. Marques, S., Zeisel, A., Codeluppi, S., van Bruggen, D., Falcão, A.M., Xiao, L., Li, H., Häring, M., Hochgerner, H. and Romanov, R.A.J.S. (2016) Oligodendrocyte heterogeneity in the mouse juvenile and adult central nervous system. *Science*, **352**, 1326-1329.
12. Pollen, A.A., Nowakowski, T.J., Shuga, J., Wang, X., Leyrat, A.A., Lui, J.H., Li, N., Szpankowski, L., Fowler, B. and Chen, P.J.N.b. (2014) Low-coverage single-cell mRNA sequencing reveals cellular heterogeneity and activated signaling pathways in developing cerebral cortex. *Nature biotechnology*, **32**, 1053-1058.
13. Tasic, B., Menon, V., Nguyen, T.N., Kim, T.K., Jarsky, T., Yao, Z., Levi, B., Gray, L.T., Sorensen, S.A. and Dolbeare, T.J.N.n. (2016) Adult mouse cortical cell taxonomy revealed by single cell transcriptomics. *Nature neuroscience*, **19**, 335-346.
14. Yan, L., Yang, M., Guo, H., Yang, L., Wu, J., Li, R., Liu, P., Lian, Y., Zheng, X., Yan, J.J.N.s. *et al.* (2013) Single-cell RNA-Seq profiling of human preimplantation embryos and embryonic stem cells. *Nature structural molecular biology*, **20**, 1131-1139.
15. Zheng, G.X., Terry, J.M., Belgrader, P., Ryvkin, P., Bent, Z.W., Wilson, R., Ziraldo, S.B., Wheeler, T.D., McDermott, G.P. and Zhu, J.J.N.c. (2017) Massively parallel digital transcriptional profiling of single cells. *Nature communications*, **8**, 1-12.
16. Domingo-Gonzalez, R., Zanini, F., Che, X., Liu, M., Jones, R.C., Swift, M.A., Quake, S.R., Cornfield, D.N. and Alvira, C.M.J.E. (2020) Diverse homeostatic and immunomodulatory roles of immune cells in the developing mouse lung at single cell resolution. *Elife*, **9**, e56890.
17. Zanini, F., Che, X., Suresh, N., Knutsen, C., Klavina, P., Xie, Y., Domingo-Gonzales, R., Jones, R.C., Quake, S.R. and Alvira, C.M.J.b. (2021) Progressive Increases in Mesenchymal Cell Diversity Modulate Lung Development and are Attenuated by Hyperoxia. *bioRxiv*.
18. Zanini, F., Che, X., Knutsen, C., Liu, M., Suresh, N., Domingo-Gonzalez, R., Dou, S.H., Jones, R.C., Cornfield, D.N. and Quake, S.R.J.b. (2021) Phenotypic diversity and sensitivity to injury of the pulmonary endothelium during a period of rapid postnatal growth. *bioRxiv*.
19. Schmidt, M.N., Winther, O. and Hansen, L.K. (2009) In Adali, T., Jutten, C., Romano, J. M. T. and Barros, A. K. (eds.), *Independent Component Analysis and Signal Separation*. Springer Berlin Heidelberg, Berlin, Heidelberg, Vol. 5441, pp. 540-547.
20. Spearman, C. (1961) *"General Intelligence" Objectively Determined and Measured*. Appleton-Century-Crofts, East Norwalk, CT, US.
21. Hyvärinen, A. and Oja, E. (2000) Independent component analysis: algorithms and applications. *Neural Networks*, **13**, 411-430.

22. Dasgupta, S. (2013) Experiments with Random Projection. *arXiv:1301.3849 [cs, stat]*.
23. Ross, D.A., Lim, J., Lin, R.-S. and Yang, M.-H. (2008) Incremental Learning for Robust Visual Tracking. *International Journal of Computer Vision*, **77**, 125-141.
24. Tenenbaum, J.B., Silva, V.d. and Langford, J.C. (2000) A Global Geometric Framework for Nonlinear Dimensionality Reduction. *Science*, **290**, 2319-2323.
25. Szubert, B., Cole, J.E., Monaco, C. and Drozdov, I. (2019) Structure-preserving visualisation of high dimensional single-cell datasets. *Scientific Reports*, **9**, 8914.
26. Schölkopf, B., Smola, A. and Müller, K.-R. (1997) In Gerstner, W., Germond, A., Hasler, M. and Nicoud, J.-D. (eds.). Springer, pp. 583-588.
27. Blei, D.M., Ng, A.Y. and Jordan, M.I. (2003) Latent Dirichlet Allocation. *Journal of Machine Learning Research*, **3**, 993-1022.
28. Lin, C.-J. (2007) Projected Gradient Methods for Nonnegative Matrix Factorization. *Neural Computation*, **19**, 2756-2779.
29. Roweis, S.T. and Saul, L.K. (2000) Nonlinear Dimensionality Reduction by Locally Linear Embedding. *Science*, **290**, 2323-2326.
30. Cichocki, A. and Phan, A.-H. (2009) Fast Local Algorithms for Large Scale Nonnegative Matrix and Tensor Factorizations. *IEICE TRANSACTIONS on Fundamentals of Electronics, Communications and Computer Sciences*, **E92-A**, 708-721.
31. Lee, D.D. and Seung, H.S. (1999) Learning the parts of objects by non-negative matrix factorization. *Nature*, **401**, 788-791.
32. Pascual-Montano, A., Carazo, J.M., Kochi, K., Lehmann, D. and Pascual-Marqui, R.D. (2006) Nonsmooth nonnegative matrix factorization (nsNMF). *IEEE Transactions on Pattern Analysis and Machine Intelligence*, **28**, 403-415.
33. Moon, K.R., van Dijk, D., Wang, Z., Gigante, S., Burkhardt, D.B., Chen, W.S., Yim, K., Elzen, A.v.d., Hirn, M.J., Coifman, R.R. *et al.* (2019) Visualizing structure and transitions in high-dimensional biological data. *Nature Biotechnology*, **37**, 1482-1492.
34. Halko, N., Martinsson, P.G. and Tropp, J.A. (2011) Finding Structure with Randomness: Probabilistic Algorithms for Constructing Approximate Matrix Decompositions. *SIAM Review*, **53**, 217-288.
35. Laurberg, H., Christensen, M.G., Plumbley, M.D., Hansen, L.K. and Jensen, S.H. (2008), *Computational Intelligence and Neuroscience*.
36. Dueck, D., Frey, B.J., Dueck, D. and Frey, B.J. (2004).
37. Amodio, M., van Dijk, D., Srinivasan, K., Chen, W.S., Mohsen, H., Moon, K.R., Campbell, A., Zhao, Y., Wang, X., Venkataswamy, M. *et al.* (2019) Exploring single-cell data with deep multitasking neural networks. *Nature Methods*, **16**, 1139-1145.
38. Kim, H. and Park, H. (2007) Sparse non-negative matrix factorizations via alternating non-negativity-constrained least squares for microarray data analysis. *Bioinformatics*, **23**, 1495-1502.
39. Zou, H., Hastie, T. and Tibshirani, R. (2006) Sparse Principal Component Analysis. *Journal of Computational and Graphical Statistics*, **15**, 265-286.
40. Li, P., Hastie, T.J. and Church, K.W. (2006). Association for Computing Machinery, pp. 287–296.
41. Belkin, M. and Niyogi, P. (2002) In Dietterich, T. G., Becker, S. and Ghahramani, Z. (eds.), *Advances in Neural Information Processing Systems 14*. MIT Press, pp. 585–591.
42. Maaten, L.v.d. and Hinton, G. (2008) Visualizing Data using t-SNE. *Journal of Machine Learning Research*, **9**, 2579-2605.
43. McInnes, L., Healy, J. and Melville, J. (2018) UMAP: Uniform Manifold Approximation and Projection for Dimension Reduction. *arXiv:1802.03426 [cs, stat]*.
44. Wang, D. and Gu, J. (2017) VASC: dimension reduction and visualization of single cell RNA sequencing data by deep variational autoencoder. *bioRxiv*, 199315.
45. Chen, S., Hua, K., Cui, H. and Jiang, R. (2019) VPAC: Variational projection for accurate clustering of single-cell transcriptomic data. *BMC Bioinformatics*, **20**, 0.
46. Pierson, E. and Yau, C. (2015) ZIFA: Dimensionality reduction for zero-inflated single-cell gene expression analysis. *Genome Biology*, **16**, 241.
47. Saelens, W., Cannoodt, R., Todorov, H. and Saeys, Y.J. (2019) A comparison of single-cell trajectory inference methods. *Nature biotechnology*, **37**, 547-554.

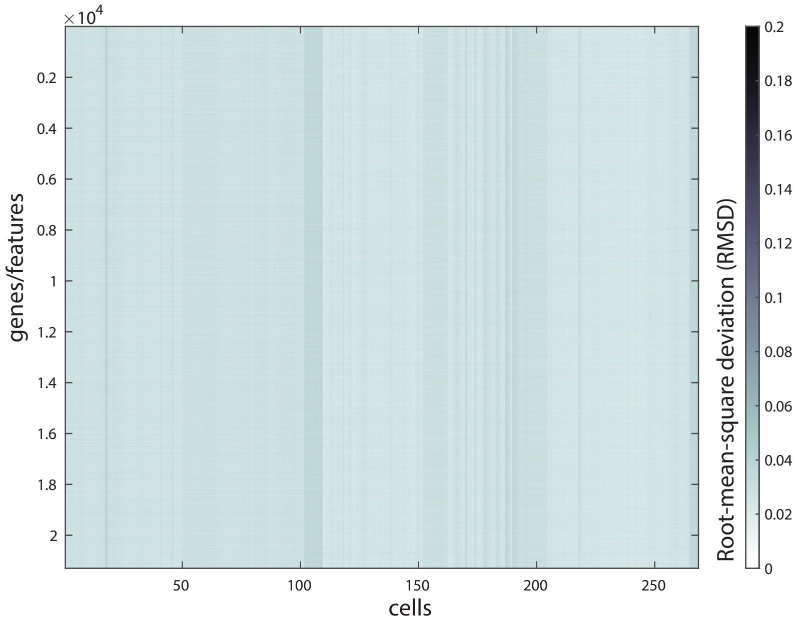

Supplementary Fig 1. The Heatmap shows root-mean-square deviations (RMSD) of scPSD-transformed gene expression after 100 random shuffles using deng reads dataset (database # 13 in Supplementary Table 1 with n = 22,431 genes/features and m = 268 cells). In order to show independency of transformation on initial ordering of features, a gene ordering was randomly picked as the 'reference order' and then the order of transcripts was shuffled 100 times prior to scPSD transformation. After applying scPSD, the transformed matrices were rearranged to unify gene ordering based on the 'reference order'. Accordingly, for each gene k in cell j, RMSD was calculated estimating the mean-square deviation of transformed gene expression compared to the corresponding expression in the reference matrix. The heatmap is therefore an nxm matrix where each element shows RMSD for individual genes k = 1, …, n across cells j = 1, … m. The minimum and maximum RMSD obtained are 2.47e-18 and 4.33e-2, respectively.

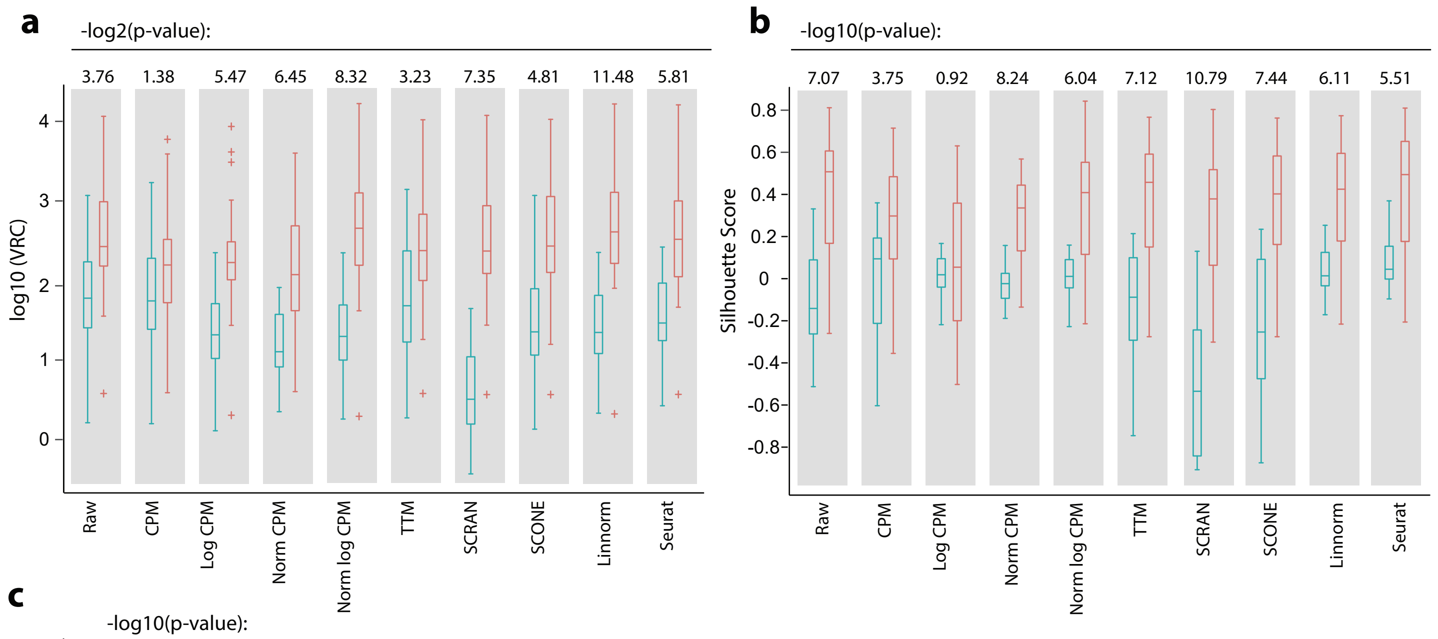

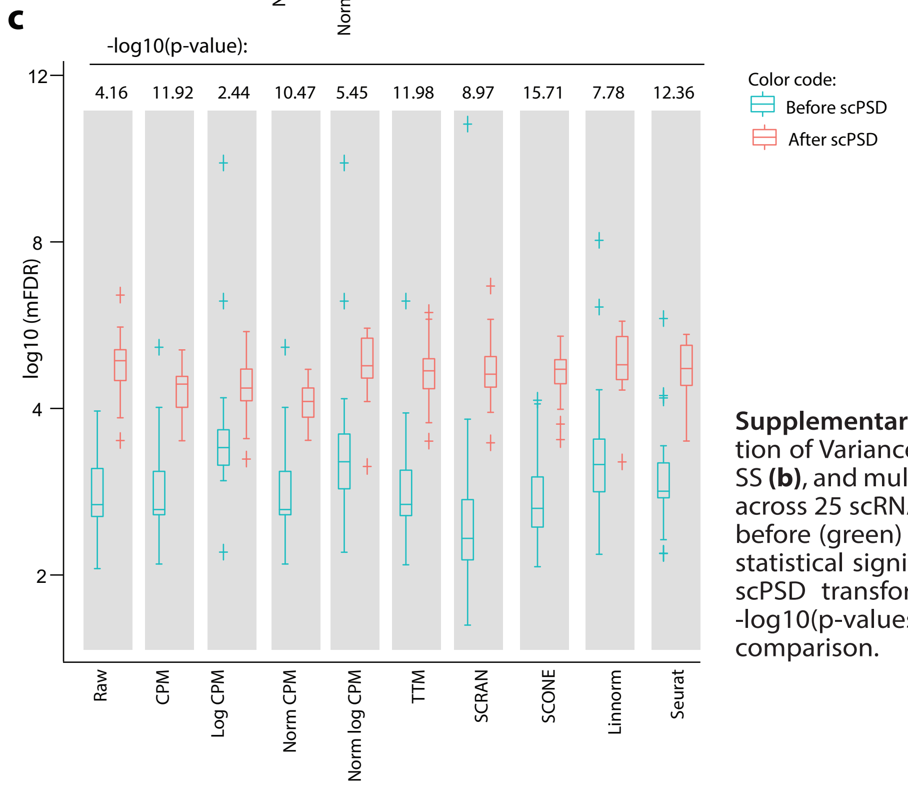

Supplementary Fig 2. Boxplots representing the distribution of Variance Ratio Criterion, VRC **(a)**, Silhouette Score, SS **(b)**, and multiclass Fisher's Discriminant Ratio, mFDR **(c)** across 25 scRNA-seq datasets (c.f. Supplementary Table 1) before (green) and after (red) scPSD transformations. The statistical significance of improvements on metrics upon scPSD transformation has been assessed using t-test; -log10(p-values) are reported on top of each pairwise comparison.

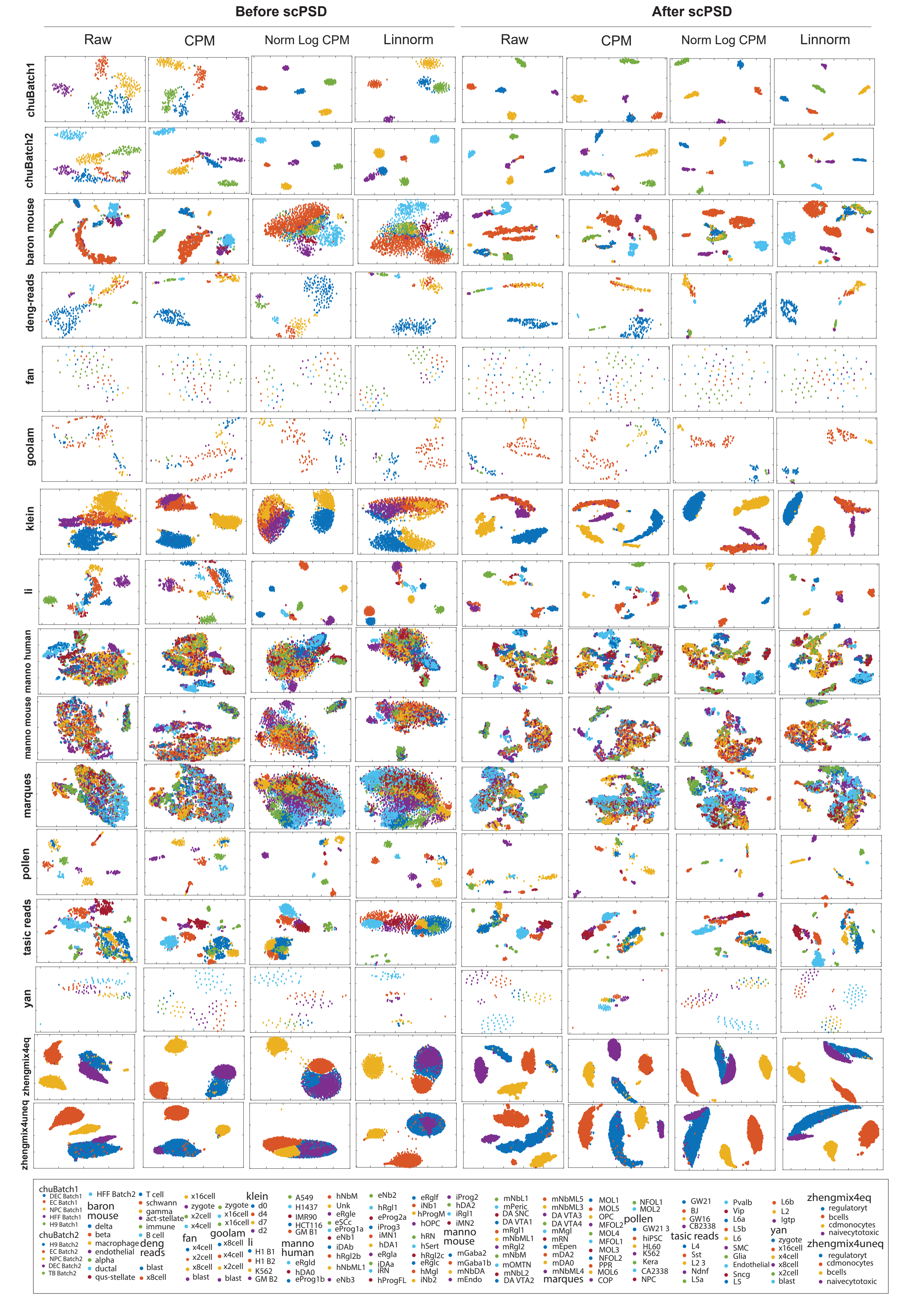

Supplementary Fig 3. 2D visualization (t-SNE dimensionality reduction) of scRNA-seq datasets (c.f. Supplementary Table 1) demonstrating visual separation of cell types upon scPSD transformation of raw or normalised data.

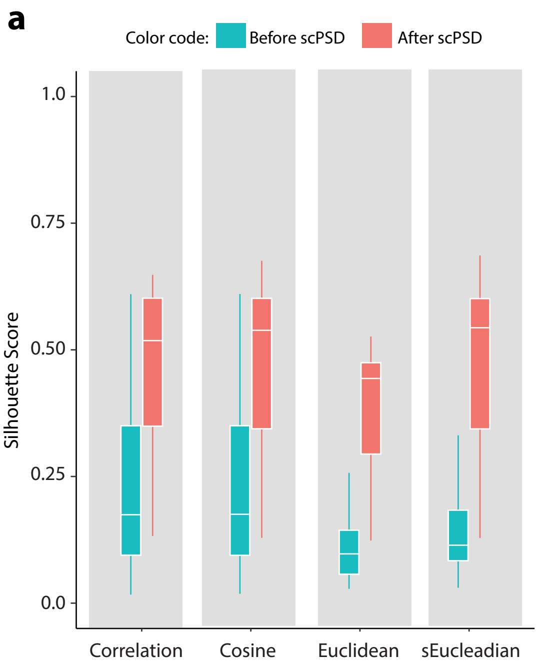

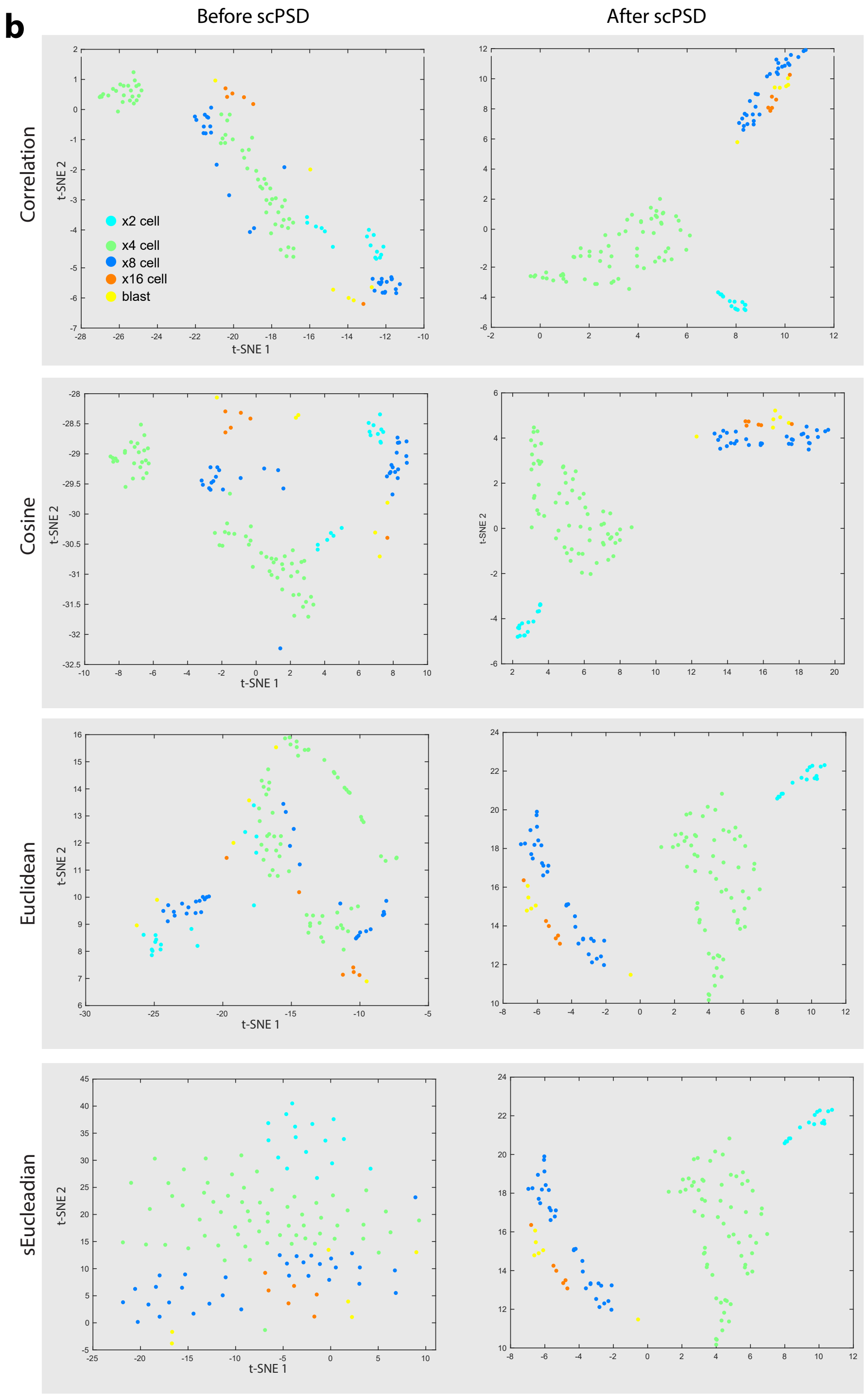

Supplementary Fig 4. Effect of distance metrics on quantitative and qualitative separation of cell types before and after scPSD transformation. Four commonly-used distance metrics including Euclidean, standardized Euclidean, one minus correlation, and one minus cosine similarity were used to estimate the separability of cell-type clusters on 14 scRNA-seq datasets (i.e., deng read, yan, tasic, pollen, marques, li, klein, goolam, fan, manno mouse, manno human, baron, zhengmix4eq, zhengmix4uneq) as detailed in Supplementary Table 1. **a,** Box plots representing the distribution of silhouette scores measuring cell-type separation based on different distance metrics across 14 datasets. **b,** Qualitative separation of clusters in mouse embryos during differentiation (goolam [7] dataset) as visualised by 2D plots after t-SNE dimensionality reduction using different distance metrics. Similar observation was obtained in other datasets that we tried; t-SNE embeddings were generated using 'approximate' method (i.e., KNN method as implemented in MATLAB).

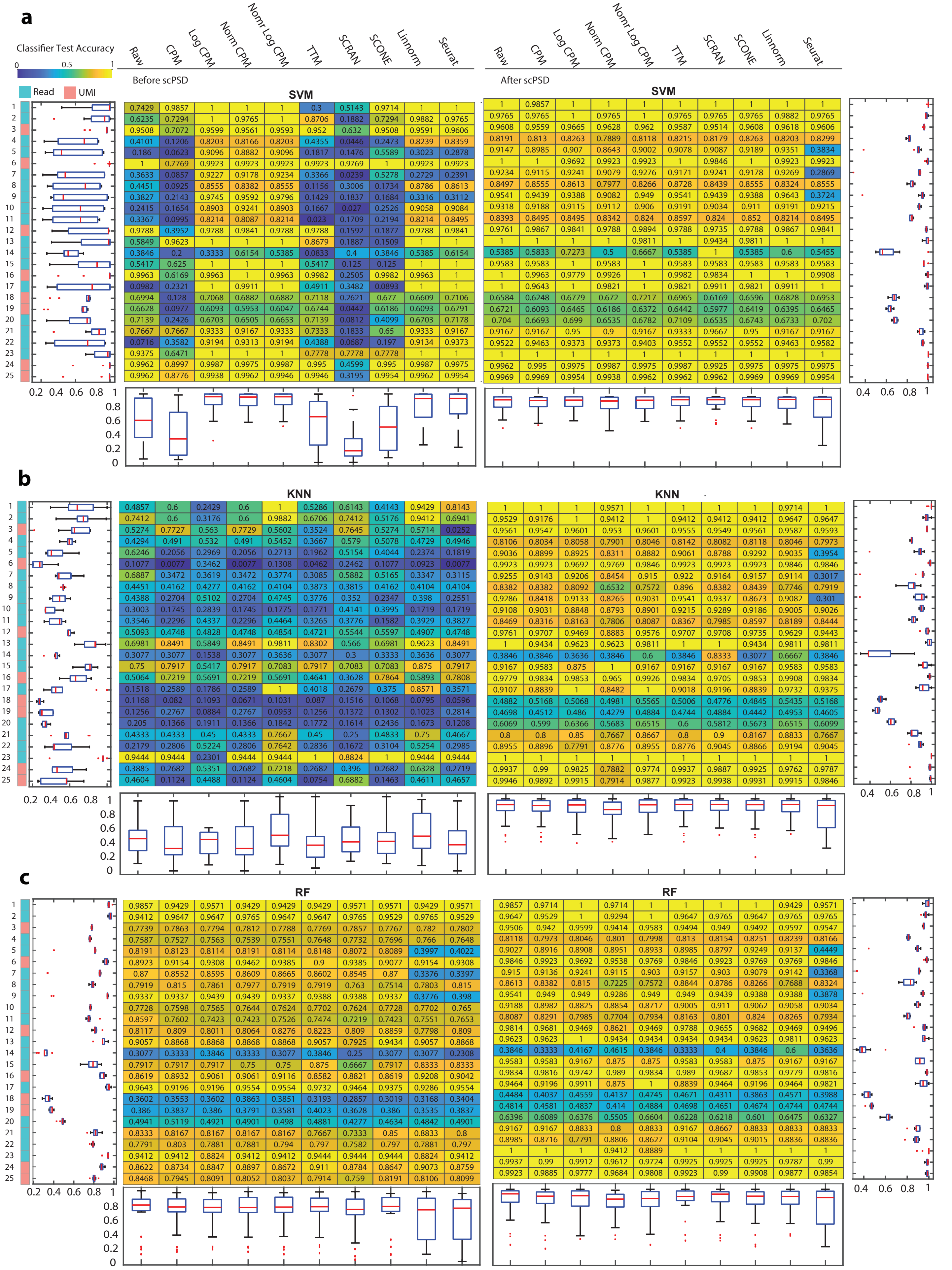

Supplementary Fig 5. Heatmaps with marginal boxplots representing cell type classification performance across each dataset before and after scPSD feature extraction (with different normalization methods used as upstream processing). Three classifiers were studied including support vector machine, SVM **(a)**, random forest, RF **(b)** and k-nearest neighbor, KNN **(c)**. For each dataset, the performance was evaluated based on the classification accuracy over a holdout test set (20% random split of a dataset)

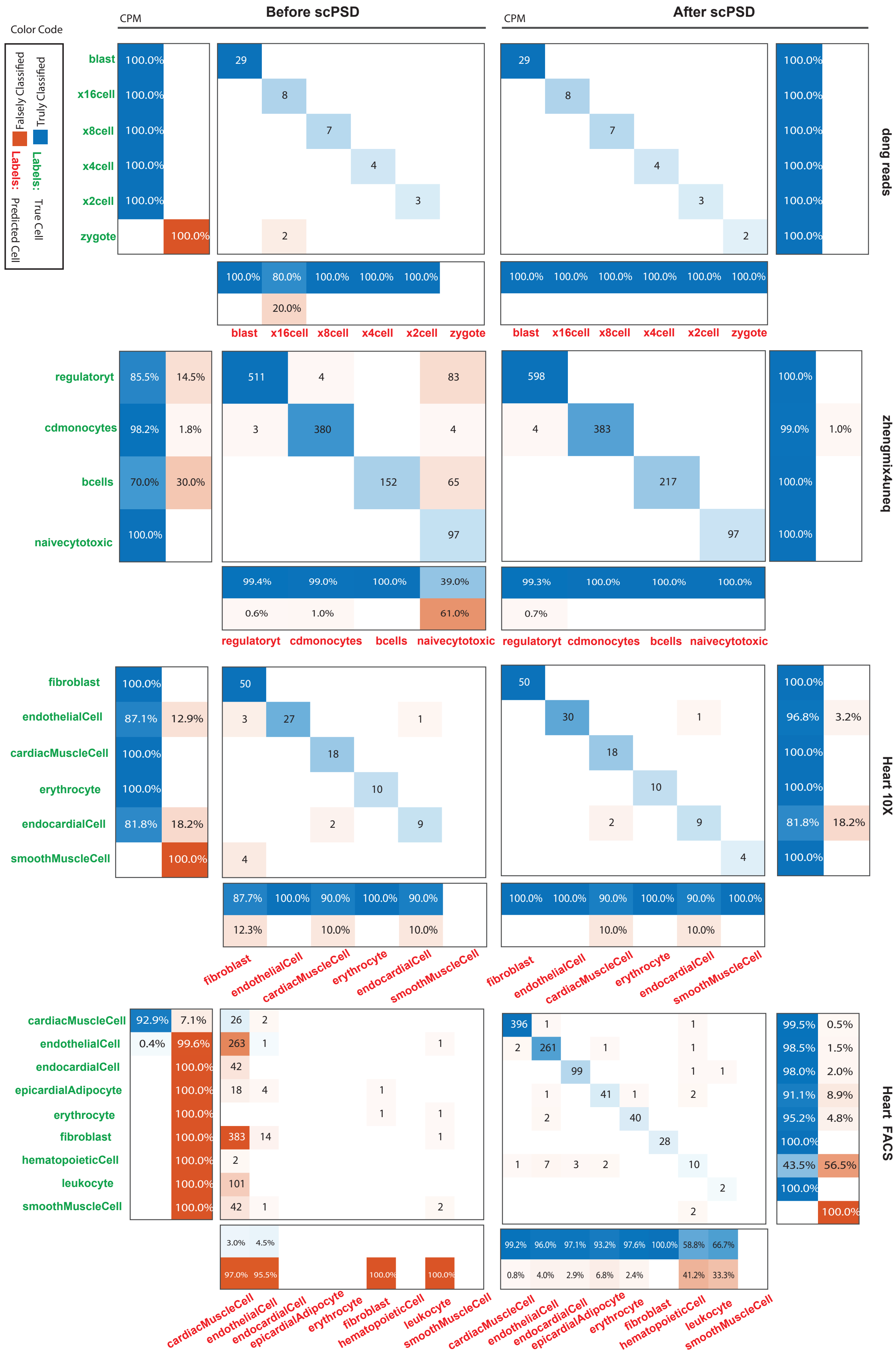

Supplementary Fig 6. Confusion matrices (CMs) detailing the performance of SVM classification on test data (randomly picked 20% of dataset). Each row of a matrix represents the instances in an actual class (green, determined based on the dataset annotations) while each column represents the instances in a predicted class (red, determined by trained SVM classifier). CMs were visualised for four datasets with comparatively reliable annotations (c.f. Supplementary Table 1) before and after scPSD transformation to assess classification performance on cell types with small populations (cell types or classes are sorted in descending order of their size). The results demonstrate the capacity of scPSD to enhance rare cell type identification. Similar results were observed on other public and in-house datasets (c.f. Fig 2).

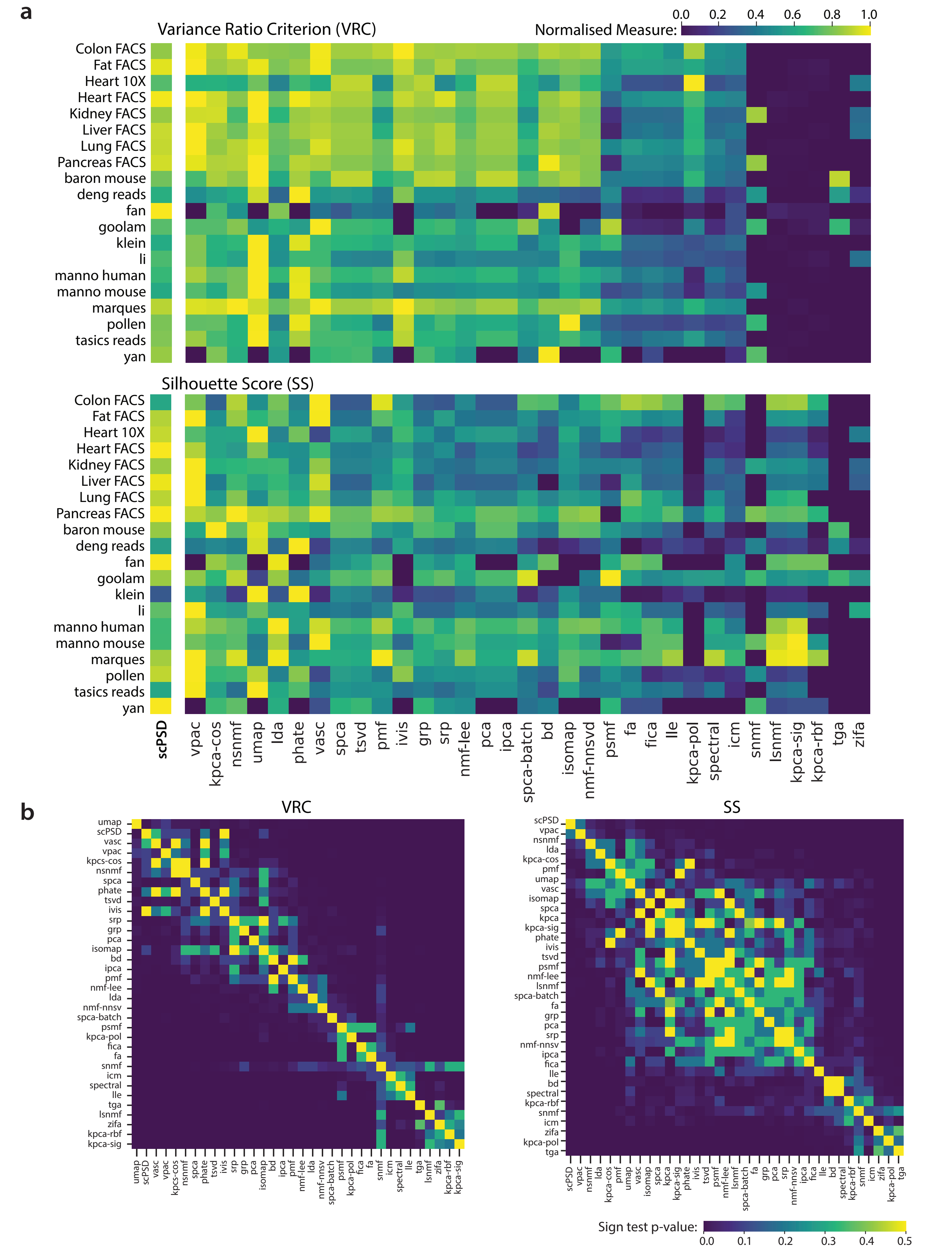

Supplementary Fig 7. (a) Heatmaps of the SS with Euclidean distance and VRC for 33 dimensionality reduction methods with embedding size of 96 (methods detailed in Supplementary Table 2). Values were standardized using min-max normalization applied per-dataset (Supplementary Table 1). Methods are orderd from left to right based on their average VRC score across all databases; scPSD shows the highest average VRC and placed at the left-most position. **(b)** Heatmaps of p-values from pairwise sign-tests comparing measures for each method across all datasets. It depicts the relative ranking of dimensionality reduction methods, as well as providing information as to which methods were roughly equivalent.

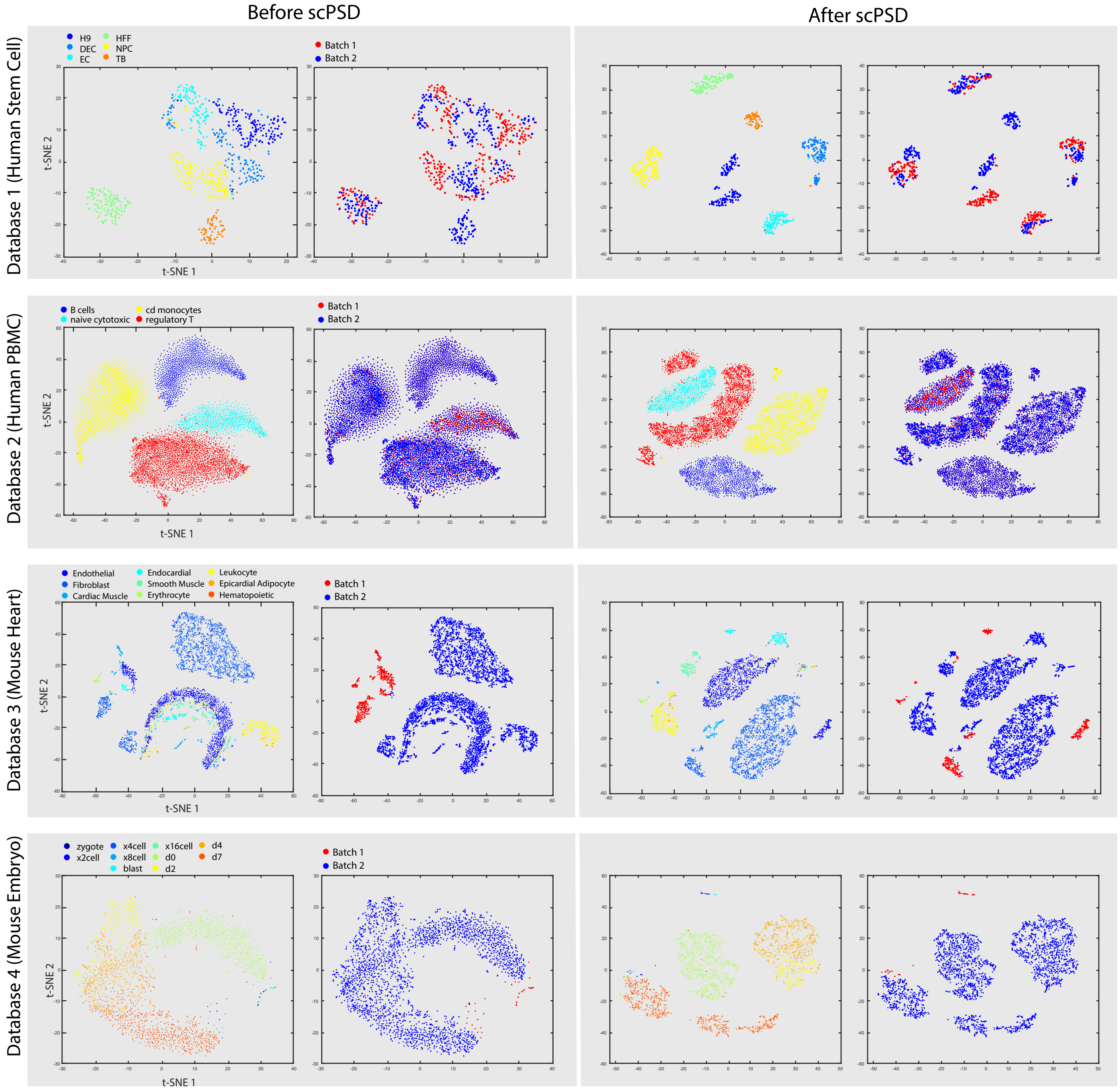

Supplementary Fig 8. The 2D t-SNE visualisation of datasets with defined batch effects based on four combined datasets (detailed in Supplementary Table 5) representing different batch-effect scenarios including batches with identical cell-types and sequencing protocols but different capturing times, batches with identical cell types but different protocols, batches containing non-identical cell types and different protocols. The t-SNE plots for each dataset (row) represent cell type separations before and after scPSD feature extraction where either cell types or batches are coloured.

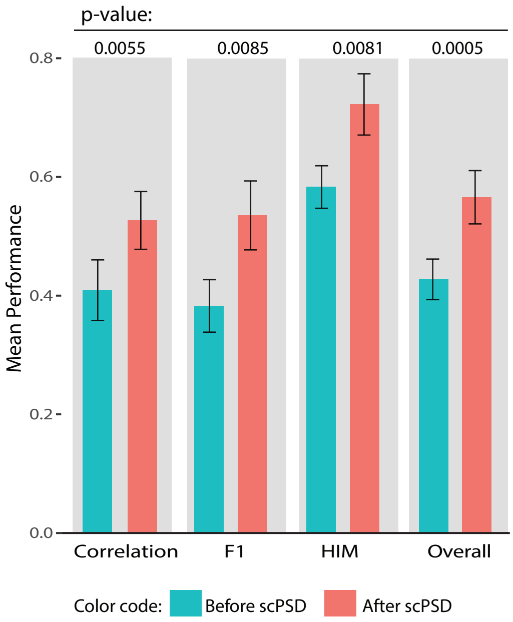

Supplementary Fig 9. Trajectory inference (TI) analysis. Bar plots representing the mean performance of minimum spanning tree (MST) in inferring the topology of 20 datasets (Supplementary Table 5) representing trajectories with different topologies (linear, bifurcation, multifurcation, and tree). The error bars represent the standard error. MST method, studied by (Saelens et al, Nature Biotechnology, 2019), is a high-performing Off-the-shelf method which performs PCA dimensionality reduction, followed by clustering using the R mclust package, and finally connects clusters using a minimum spanning tree algorithm. The performance metrics include 1) HIM which measures the topological similarity 2) F1 between branch assignments, 3) correlation between geodesic distances, and 4) overall score computed as geometric mean of the three metrics (c.f. Saelens et al for details of metrics). P-value of paired t-test on top of the plot shows the significance of comparison before vs after scPSD transformation. for each metric. This initial result is not generalisable to any TI method and the effect of scPSD transformation on other well-preforming TI methods requires further investigation.
